# Phosphatidylserine exposure is required for ADAM17 sheddase function

**DOI:** 10.1038/ncomms11523

**Published:** 2016-05-10

**Authors:** Anselm Sommer, Felix Kordowski, Joscha Büch, Thorsten Maretzky, Astrid Evers, Jörg Andrä, Stefan Düsterhöft, Matthias Michalek, Inken Lorenzen, Prasath Somasundaram, Andreas Tholey, Frank D. Sönnichsen, Karl Kunzelmann, Lena Heinbockel, Christian Nehls, Thomas Gutsmann, Joachim Grötzinger, Sucharit Bhakdi, Karina Reiss

**Affiliations:** 1 Department of Dermatology, University of Kiel, Schittenhelmstrasse 7, Kiel 24105, Germany; 2 Arthritis and Tissue Degeneration Program, Hospital for Special Surgery at Weill Medical College of Cornell University, New York, New York 10021, USA; 3 Hamburg University of Applied Science, Ulmenliet 20, Hamburg 21033, Germany; 4 Institute of Biochemistry, University of Kiel, Olshausenstrasse 40, Kiel 24098, Germany; 5 Division of Systematic Proteome Research and Bioanalytics, Institute for Experimental Medicine, University of Kiel, Kiel 24105, Germany; 6 Otto Diels Institute for Organic Chemistry, University of Kiel, Kiel 24118, Germany; 7 Physiological Institute, University of Regensburg, Universitätsstrasse 31, Regensburg 93053, Germany; 8 Forschungszentrum Borstel, Leibniz-Zentrum für Medizin and Biowissenschaften, Borstel 23845, Germany

## Abstract

ADAM17, a prominent member of the ‘Disintegrin and Metalloproteinase' (ADAM) family, controls vital cellular functions through cleavage of transmembrane substrates. Here we present evidence that surface exposure of phosphatidylserine (PS) is pivotal for ADAM17 to exert sheddase activity. PS exposure is tightly coupled to substrate shedding provoked by diverse ADAM17 activators. PS dependency is demonstrated in the following: (a) in Raji cells undergoing apoptosis; (b) in mutant PSA-3 cells with manipulatable PS content; and (c) in Scott syndrome lymphocytes genetically defunct in their capacity to externalize PS in response to intracellular Ca^2+^ elevation. Soluble phosphorylserine but not phosphorylcholine inhibits substrate cleavage. The isolated membrane proximal domain (MPD) of ADAM17 binds to PS but not to phosphatidylcholine liposomes. A cationic PS-binding motif is identified in this domain, replacement of which abrogates liposome-binding and renders the protease incapable of cleaving its substrates in cells. We speculate that surface-exposed PS directs the protease to its targets where it then executes its shedding function.

## Abstract (web summary)

ADAM17 is a member of the ‘Disintegrin and Metalloproteinase' family of proteases, that cleaves transmembrane substrates from the surfaces of cells. Here the authors show that surface exposure of phosphatidylserine is required for ADAM17 sheddase activity, possibly by directing the protease to its substrates.

## Graphical abstract

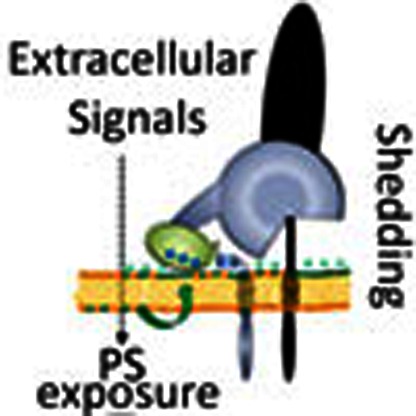

The evolutionarily conserved ADAM (a disintegrin and metalloprotease) family of cell-bound proteinases regulates a plethora of biological functions via cleavage of transmembrane substrates^1^. ADAM17, originally discovered as the TNF-α-converting (cleaving) enzyme, has emerged as a pre-eminent member of this family^2^,^3^,^4^. More than 80 ADAM17 targets have been identified to date, prominent among which are cytokines, cell surface receptors and adhesion molecules^5^. Although several ‘preferential' substrates such as L-selectin^4^,^6^,^7^,^8^ or tumour necrosis factor receptor type 1 (TNFR1)^9^,^10^ exist, some others are shared with the closely related protease ADAM10 (ref. 11). Substrate cleavage sites contain no consensus sequence and, remarkably, they have only one feature in common: they are located very near to the surface of the lipid bilayer^12^.

ADAM17 regulates cell growth through the liberation of epidermal growth factor receptor (EGFR) ligands and subsequent activation of ERK1/2 signalling^13^,^14^,^15^. Although ADAM17 is the major sheddase of transforming growth factor-α (TGF-α), amphiregulin, epiregulin and heparin-binding EGF, ADAM10 is predominantly involved in the release of EGF and betacellulin^14^.

In the mouse, deletion of ADAM17 leads to embryonic lethality due to uncorrectable developmental defects ensuing from disrupted EGFR signalling^4^. In humans, ADAM17 deficiency results in severe inflammatory skin and bowel disease, underlining its important role for epithelial cell homeostasis^16^,^17^.

Regulation of ADAM function occurs at many levels. Exit from the endoplasmic reticulum requires interaction with inactive members of the Rhomboid family of intramembrane serine proteinases (iRhom1 and iRhom2)^18^,^19^. During ADAM17 maturation, the prodomain is removed by pro-protein convertases such as furin^20^. A sorting protein named phosphofurin acidic cluster sorting protein 2 reportedly regulates ADAM17 trafficking and diverts the enzyme away from degradation pathways^21^. On the cell surface, sheddase activity can be rapidly induced by remarkably diverse stimuli including protein kinase C (PKC) activators, purine 2 (P2) receptor agonists, fibroblast growth factor 7 (FGF7), Ca^2+^ ionophores and membrane perturbants^22^,^23^,^24^,^25^. Inhibition of the responsible intracellular signalling cascades abolishes these effects. How the very heterogeneous pathways triggered by these agents should convene to activate one and the same protease at the extracellular membrane surface has remained a fascinating enigma in cell biology. The fact that the cytoplasmic domain of ADAM17 is not required for sheddase activation^9^,^22^,^23^,^24^,^26^ renders the mystery yet more perplexing.

Independent of any stimuli, ADAM17 sheddase activity is enhanced in cells undergoing apoptosis^27^. A hallmark of these cells is the breakdown of phospholipid asymmetry with a remarkable increase of phosphatidylserine (PS) exposure in the external membrane leaflet^28^. Two major processes apparently contribute to this phenomenon. First, PS and phosphatidylethanolamine passively translocating to the external leaflet are normally returned to the inner leaflet through the action of ATP-dependent ‘flippases'. Second, events such as Ca^2+^ elevation and apoptosis are accompanied by the activation of scramblases, proteins that nonspecifically and bidirectionally translocate phospholipids between the outer and inner leaflets of the plasma membrane^29^. The molecules that flip and scramble have proved very difficult to characterize. In recent times, two membrane proteins have been identified as scramblases: transmembrane protein 16F (TMEM16F)^30^ and Xk-related protein 8 (Xkr8)^31^. Apoptosis is accompanied by caspase-dependent Xkr8 activation and irreversible flippase inactivation. In contrast, TMEM16F is responsible for reversible PS exposure that occurs in response to cytoplasmic Ca^2^^+^ elevation^28^.

PS externalization has generally been considered to mainly serve as an ‘eat-me' signal for apoptotic cells. However, it is readily conceivable that the anionic phospholipid may itself regulate cellular functions. Elliott *et al*.^32^ showed that rapid PS translocation and release of the ADAM17 substrate L-selectin occurs in cells stimulated with P2 receptor agonists independent of any apoptotic events.

Transient PS exposure has been reported not only in the context of purinergic receptor stimulation, but also in conjunction with PKC activation^33^ and cytosolic Ca^2+^ elevation^30^. It therefore occurred to us that translocation of PS to the outer membrane leaflet might represent the penultimate event common to all routes of ADAM17 activation.

Here we present evidence in support of this hypothesis. In diverse cell models, ADAM17 sheddase activity correlates with PS exposure. Substrate cleavage is inhibited by soluble phosphorylserine but not by phosphorylcholine. A cationic PS-binding motif is identified in the membrane proximal domain (MPD) of ADAM17. When this motif is exchanged, the resulting ADAM17 mutant retains its peptidolytic activity, yet physiological sheddase function is not detectable. We advance the hypothesis that the negatively charged PS head group interacts with cationic amino acid clusters present within the MPD of ADAM17. This interaction seems to be necessary for the protease to fulfil its sheddase function.

## Results

### ADAM17 stimuli provoke PS exposure

**Figure 1:**
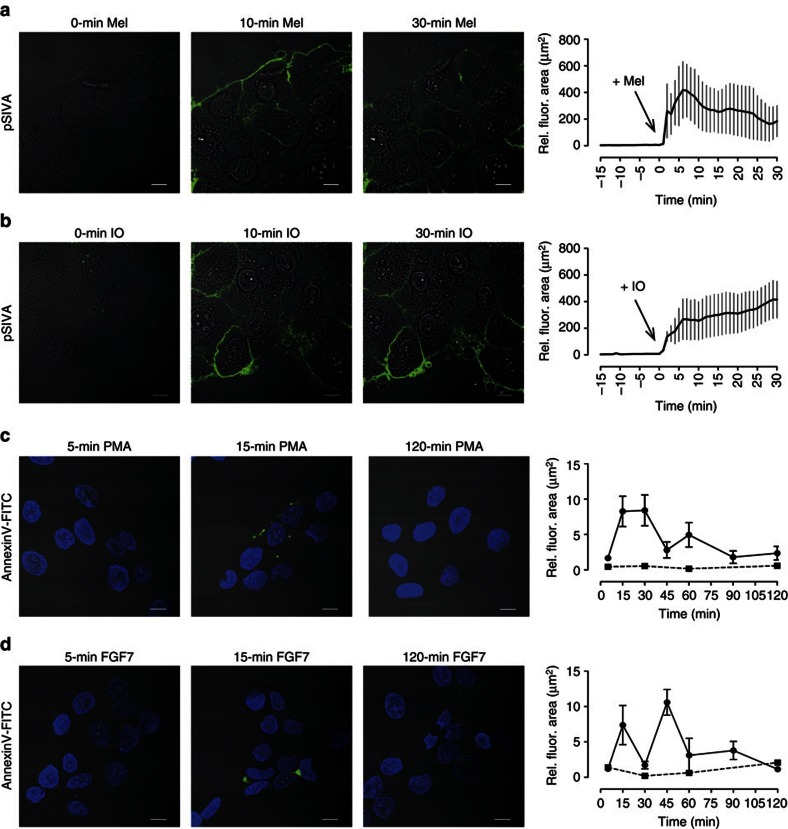
ADAM17 stimuli induce PS exposure. (**a**,**b**) HaCaT keratinocytes were stimulated with melittin (Mel, 1 μM) or IO (1 μM) and analysed for PS exposure via live-cell imaging using the polarity-sensitive PS-binding dye pSIVA. The mean fluorescence area was quantified for statistical analysis (Mel: *n*=5; ±s.e.m.; IO: *n*=3; ±s.e.m.). (**c**,**d**) HaCaTs were stimulated with PMA (300 ng ml^−1^) or FGF7 (100 ng ml^−1^) and stained with Annexin V–FITC after the indicated time points. The mean fluorescence area was quantified for statistical analysis (*n*⩾3; ±s.e.m.). Unstimulated cells were used as control (squares). Scale bars, 10 μm.

The first experiments were conducted to examine whether common ADAM17 stimuli would regularly induce PS exposure in human HaCaT keratinocytes. It was found that the potent ADAM17 activators melittin^25^ and Ca^2+^ ionophore ionomycin (IO) both provoked massive PS externalization that could be followed on a real-time basis with the use of pSIVA (Annexin XII). The cell-impermeable, polarity-sensitive agent is added to the culture medium. It displays fluorescence only when bound to lipid bilayers and thus faithfully reports the presence of PS that is exposed in the outer membrane leaflet^34^ (Fig. 1a,b). Melittin induced rapid PS exposure spreading over the entire cell surface that reached a maximum in 5–10 min and decreased thereafter (Fig. 1a and Supplementary Movie 1). PS exposure provoked by IO was yet more rapid and remained sustained over the time of observation (Fig. 1b and Supplementary Movie 2). PS externalization induced by the PKC activator phorbol 12-myristate 13-acetate (PMA) and the physiologic ADAM17 activator FGF7 was less pronounced and conventional staining with fluorescent-labelled Annexin V was employed because of higher sensitivity (Fig. 1c,d and Supplementary Fig. 1). Several waves of discrete, punctate staining became detectable over an extended period of 90 min. The oscillating nature of PS exposure was particularly conspicuous with FGF7 (Fig. 1d and Supplementary Fig. 1). The differences in kinetics and localization of surface-exposed PS possibly reflect the different signalling pathways triggered by the respective stimuli. The impression was gained that patterns of PS exposure also exhibited a degree of cell-type specificity. Stimulation of endothelial cells with IO led to an Annexin-staining pattern that appeared distinct from that observed in HaCaT keratinocytes (Supplementary Movie 3).

### Inhibitors of ADAM17-mediated shedding blunt PS exposure

Shedding of EGFR ligands is differentially induced by various stimuli. For example, FGF7 induces EGFR activation in keratinocytes via cleavage of heparin-binding EGF^24^, whereas TGF-α release remains hardly detectable (Supplementary Fig. 2). Furthermore, prolonged times are usually required for levels of shed substrates to become measurable in keratinocytes. Thus, TGF-α release can only serve as indication for ADAM17 activation and not provide a direct correlation. By contrast, the assessment of ERK1/2 activation provides an attractive option for a faster readout. Indeed, it was found that all stimuli induced ERK1/2 phosphorylation that was inhibited by the EGFR-blocking antibody Cetuximab, by the metalloprotease inhibitor marimastat (MM) and, to a large extent, by the ADAM17 inhibitory antibody D1 (ref. 35) (Supplementary Fig. 3). PMA, IO and melittin all provoke the release of TGF-α and this substrate was additionally quantified in the respective experiments.

**Figure 2:**
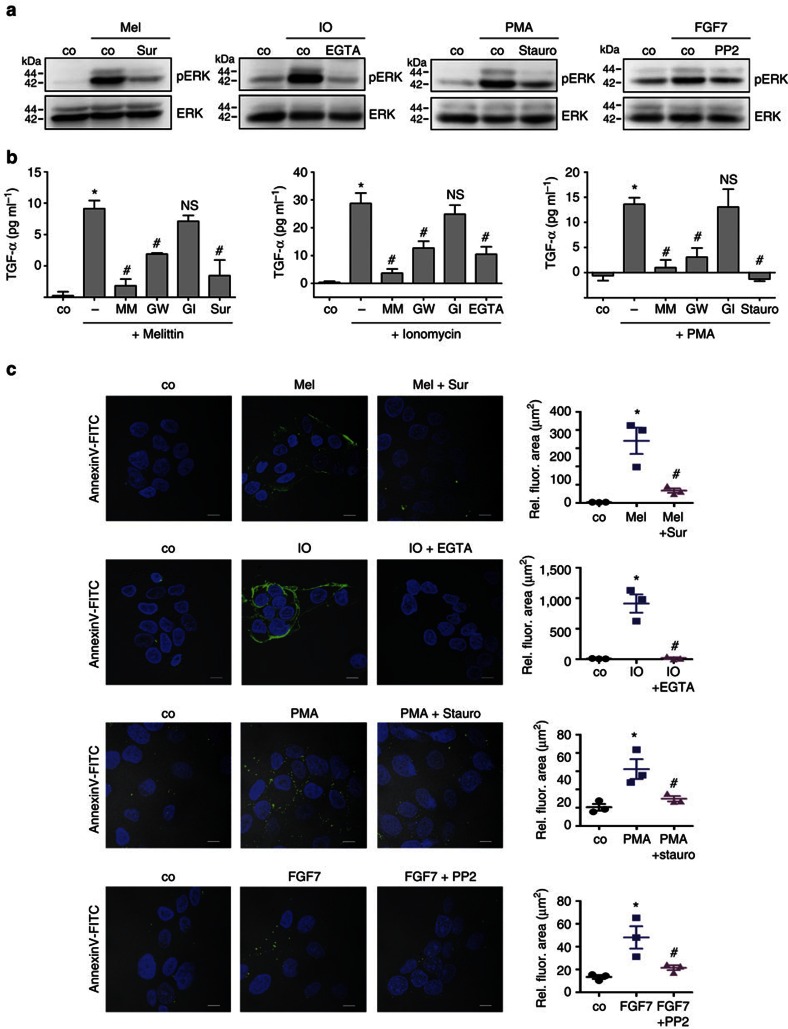
ADAM17 inhibitors abrogate PS exposure. HaCaT keratinocytes were stimulated with melittin (Mel, 1 μM), IO (1 μM), PMA (300 ng ml^−1^) or FGF7 (100 ng ml^−1^) in the presence or absence of P2 receptor inhibitor suramin (Sur, 50 μM), EGTA (10 mM), PKC inhibitor staurosporine (Stauro, 1 μM) or Src-kinase inhibitor (PP2, 10 μM). (**a**) pERK1/2 analyses were undertaken as readout for ADAM17-dependent EGFR transactivation 15 min after stimulation. Representative western blottings show abrogation of ERK1/2 activation in the presence of the inhibitors. Total ERK1/2 immunoblots are included as control. (**b**) The supernatant was analysed for soluble TGF-α by enzyme-linked immunosorbent assay (ELISA) 30 min after stimulation. All stimuli significantly increased soluble TGF-α shedding (*n*=3–6; ±s.e.m.; *P<0.05). Stimulation inhibitors, broad spectrum metalloprotease inhibitor MM (10 μM), ADAM17/10 inhibitor (GW, 2 μM) but not ADAM10 inhibitor GI (2 μM) significantly reduced TGF-α shedding (*n*=3–6; ±s.e.m.; ^#^*P*<0.05). NS, nonsignificant. (**c**) Cells were stained with Annexin V–FITC 15 min after stimulation in the presence or absence of the respective inhibitors. Representative images are shown. The mean fluorescence area was quantified for statistical analysis. *A significant increase compared with untreated controls (*n*=3; ±s.e.m; *P<0.05). ^#^Significant decrease compared with stimulated cells. Data were analysed by one-way analysis of variance and Bonferroni multiple comparison *post hoc* test. Scale bars, 10 μm.

The question followed whether inhibition of sheddase activity would be mirrored by lack of PS exposure. Cells were exposed to the stimuli in the absence or presence of respective inhibitors. As shown in Fig. 2a,b, the P2R antagonist suramin, the calcium chelator EGTA, the PKC inhibitor staurosporine and the Src-kinase inhibitor PP2 all suppressed EGFR-dependent ERK1/2 activation. Release of TGF-α was reduced accordingly. The latter was inhibited by MM and the ADAM17/ADAM10 inhibitor GW, but not by the ADAM10 inhibitor GI. Conspicuously, blunted ADAM17 activation was in every case associated with the absence of PS externalization (Fig. 2c).

### Induction of substrate release is blunted in PSA-3 cells

**Figure 3:**
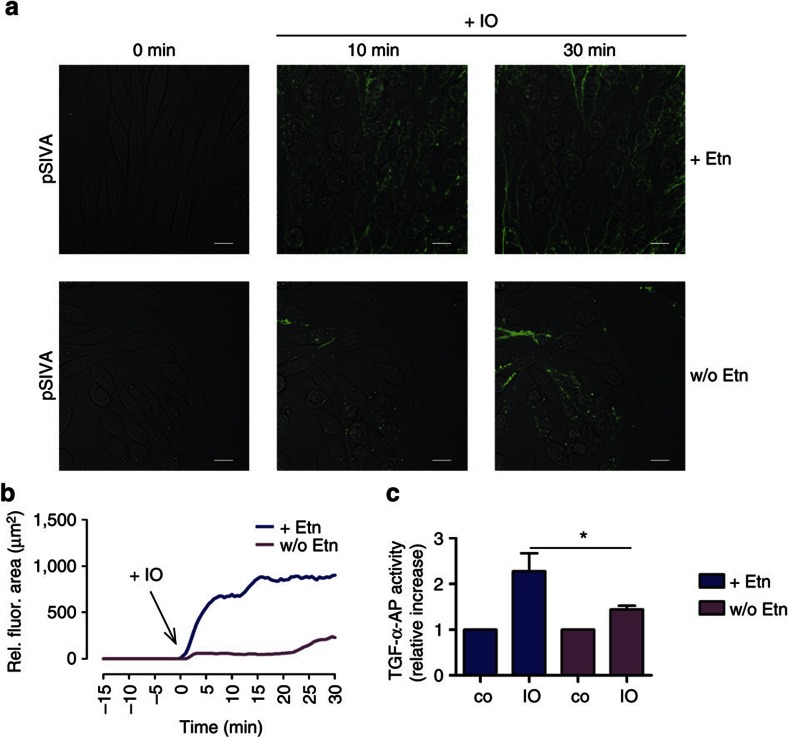
ADAM17 activation depends on PS exposure. PSA-3 cells were either cultured in the presence of ethanolamine (+Etn) or starved (w/o Etn) before IO (1 μM) stimulation. (**a**) PS exposure was determined by pSIVA live-cell imaging. Representative images are shown. Scale bars, 10 μm. (**b**) The mean fluorescence area of one representative of three independent experiments was quantified over time. (**c**) The release of transfected AP-tagged TGF-α was used as readout for ADAM17 activity. *A significant increase compared with control (*n*=6; ±s.e.m.; *P<0.05). Data were analysed by one-way analysis of variance and Bonferroni multiple comparison *post hoc* test.

Mammalian cells contain two PS synthases (PSS). PSS1 synthesizes PS from phosphatidylcholine (PC), while PSS2 synthesizes PS from phosphatidylethanolamine. PSA-3 cells are a mutant CHO-K1 cell line defective in PSS1 activity. PS levels decrease when the cells are cultured in the absence of exogenous PS and the reduced PS content of the cells can be normalized by culturing in the presence of ethanolamine^36^. In the next experiments, PSA-3 cells were transfected with alkaline phosphatase (AP)-tagged TGF-α to provide a common target for ADAM17. Cells were cultured either in medium supplemented with fetal calf serum (FCS) and ethanolamine or in medium supplemented only with dialysed FCS for 72 h. Cells were then transfected and after 24 h stimulated with IO and analysed by pSIVA live-cell imaging (Fig. 3a,b). Inhibitor experiments confirmed that ADAM17 was responsible for the IO-induced TGF-α release in these cells (Supplementary Fig. 4). As shown in Fig. 3a,b (and Supplementary Movies 4 and 5), cells cultured in the presence of ethanolamine responded with PS exposure and abundant release of TGF-α (Fig. 3c), while cells deprived of their PS source exposed little PS and shed little TGF-α.

### PS exposure is required for shedding accompanying apoptosis

**Figure 4:**
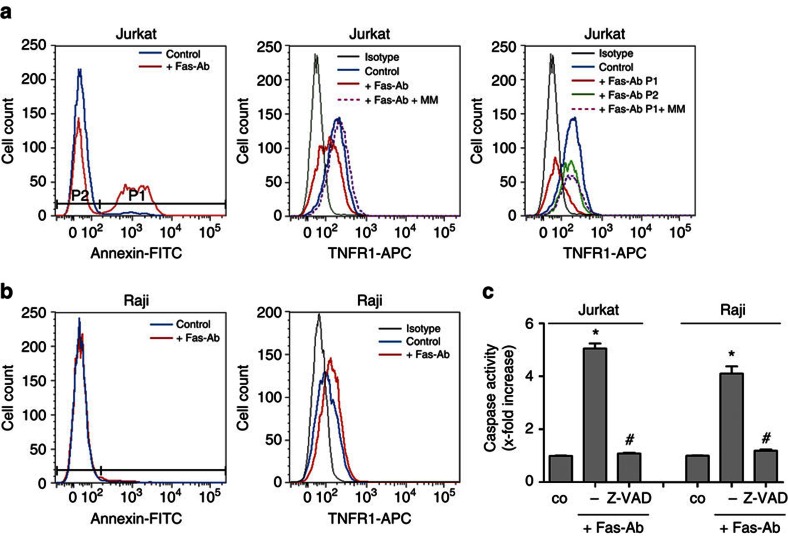
Apoptosis only activates ADAM17 sheddase activity in the presence of PS exposure. Jurkat cells (**a**) and Raji lymphoma cells (**b**) were stimulated with agonistic anti-Fas antibody (Fas-Ab, 500 ng ml^−1^) in the presence or absence of MM (10 μM) for 2 h. Flow cytometric analysis of PS exposure (Annexin V–FITC staining) and TNFR1 expression was performed. Fas stimulation induced PS externalization (**a**, left panel) and TNFR1 shedding (**a**, middle and right panel) in Jurkat cells but not in Raji cells (**b**). The PS-positive cell population (P1) nearly completely lost TNFR1 (**a**, right panel, red line), while the PS negative population (P2) was hardly affected (**a**, right panel, green line). MM (**a**, dotted purple line) rescued the induced shedding. Representative results of three independent experiments are shown. (**c**) Caspase-3 cleavage was analysed in parallel in the presence or absence of caspase inhibitor Z-VAD (10 μM). Caspase activation was significantly induced (*n*=3; ±s.e.m.; *P<0.05) in both cell types. Z-VAD significantly reduced caspase activation (*n*=3; ±s.e.m.; ^#^P<0.05). Data were analysed by one-way analysis of variance and Bonferroni multiple comparison *post hoc* test.

PS exposure is the hallmark of apoptosis and apoptosis is a trigger of ADAM-mediated substrate release^27^,^37^,^38^. However, human Raji lymphoma cells undergoing apoptosis expose minimal amounts of PS^39^,^40^ due to low expression of the caspase-activated scramblase Xkr8 (ref. 31). A unique possibility thus arose to examine whether PS externalization and ADAM17-mediated shedding were tightly coupled events. TNFR1 is predominantly cleaved by ADAM17 (ref. 10) and present on Jurkat and Raji cells. Exposure to agonistic anti-Fas antibody (Fas-Ab) resulted in PS externalization in Jurkat (Fig. 4a, left panel) but not in Raji cells (Fig. 4b, left panel), despite induction of apoptosis (Fig. 4c). Strikingly, TNFR1 shedding accompanying apoptosis occurred only in Jurkat cells (Fig. 4a, middle and right panel). The PS-positive cell population (P1) showed a nearly complete loss of the TNFR1, which was rescued by MM (Fig. 4a) and the ADAM17 inhibitory antibody D1 (Supplementary Fig. 5). In contrast, the PS-negative cells (P2) were barely affected. These data indicate that activation of the apoptotic signalling cascade itself is not the trigger of ADAM17 activation and underline the importance of PS exposure for the shedding event.

### Defective PS externalization results in reduced shedding

Blood cells from Scott syndrome patients present a counterpart to Raji cells. These cells have a missense mutation in TMEM16F, the scramblase that is responsible for rapid PS exposure in response to Ca^2+^ elevation in blood cells^30^,^41^. In contrast, PS exposure provoked via other pathways including Fas-triggered apoptosis remains undisturbed. The major consequence of the defect is therefore relatively restricted to platelet malfunction, which underlies the clinical manifestation of this rare bleeding disorder.

ADAM17 is a major sheddase of L-selectin on leukocytes^6^,^7^,^8^, which can conveniently be assessed by flow cytometry. Scott cells showed the same cell surface expression of ADAM17 as control cells, so that comparable basic conditions for assessment of shedding were given (Supplementary Fig. 6a).

**Figure 5:**
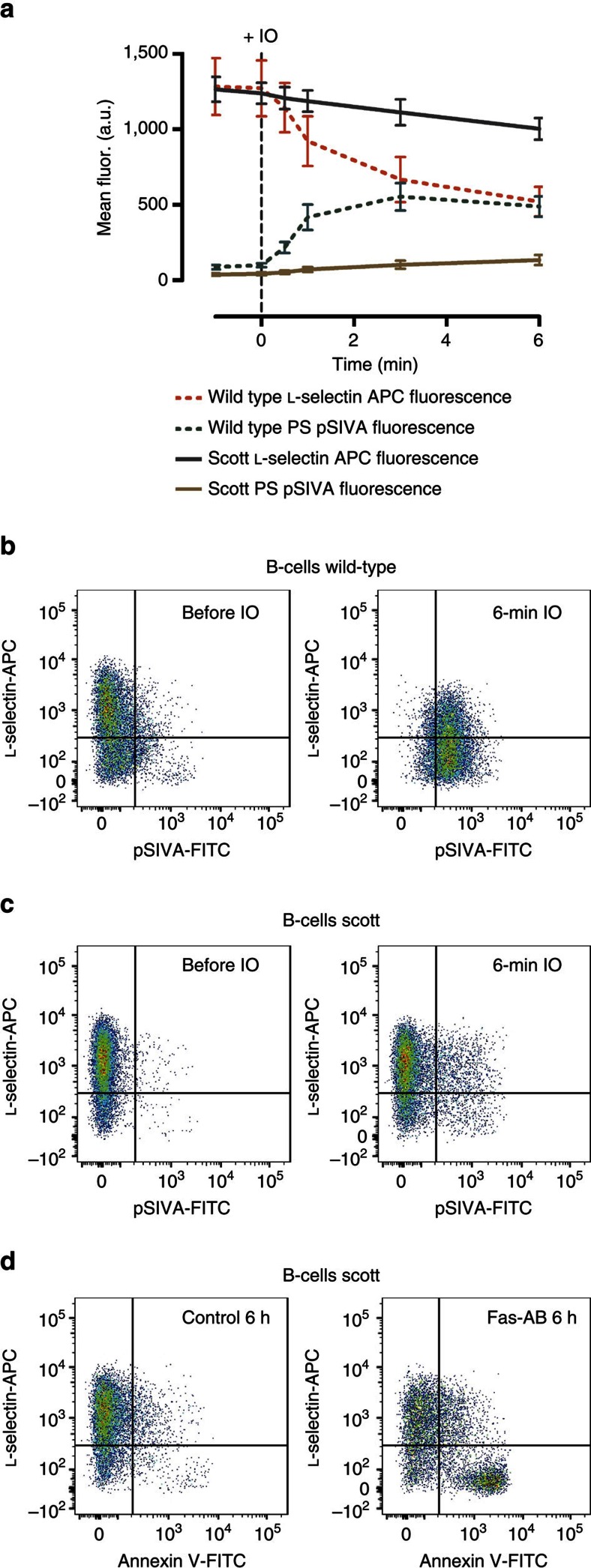
Defective PS externalization abolishes ADAM17-mediated shedding. Normal B cells and B cells from Scott syndrome patients were monitored by flow cytometry for induction of PS exposure (pSIVA) and L-selectin shedding. (**a**) Time course of the mean fluorescence after IO (2 μM) stimulation over 6 min. IO concomitantly induced PS externalization and loss of L-selectin in WT cells (**a**,**b**), but not in Scott patient cells (**a**,**c**). Mean fluorescence data in **a** are presented as mean±s.e.m. from five independent experiments. Representative pseudocolour plots of normal B cells (**b**) and Scott patient cells (**c**) before and 6 min after addition of IO are shown. (**d**) Fas-antibody (Fas-Ab, 500 ng ml^−1^, 6 h)-induced apoptosis led to pronounced PS exposure and concomitant L-selectin loss in Scott cells.

When calcium ionophore was applied to control B cells, flow cytometric analyses revealed rapid PS exposure that was parallelled by loss of L-selectin from the cell surface (Fig. 5a,b and Supplementary Fig. 6b). The temporal congruence between the two events, revealed here at the single-cell level, was impressive. L-selectin shedding was abrogated in the presence of MM and strongly reduced by the ADAM17 inhibitory antibody D1 (Supplementary Fig. 6c). Intriguingly, Scott lymphocytes responded to the ionophore neither with PS exposure nor with shedding of the ADAM17 substrate (Fig. 5a,c). However, and in telling contrast, exposure to Fas antibody led to PS exposure on these cells and to concomitant loss of L-selectin (Fig. 5d), as observed in normal lymphocytes (Supplementary Fig. 6d).

### Soluble phosphorylserine suppresses sheddase function

Should surface-exposed PS be influencing ADAM17 function through interaction of the phospholipid head group with the protease, this would most probably occur at the extracellular membrane surface and might be competitively inhibitable by soluble phosphorylserine (OPS, *O*-phospho-L-serine). This question was addressed in different cell lines for different ADAM17 substrates.

**Figure 6:**
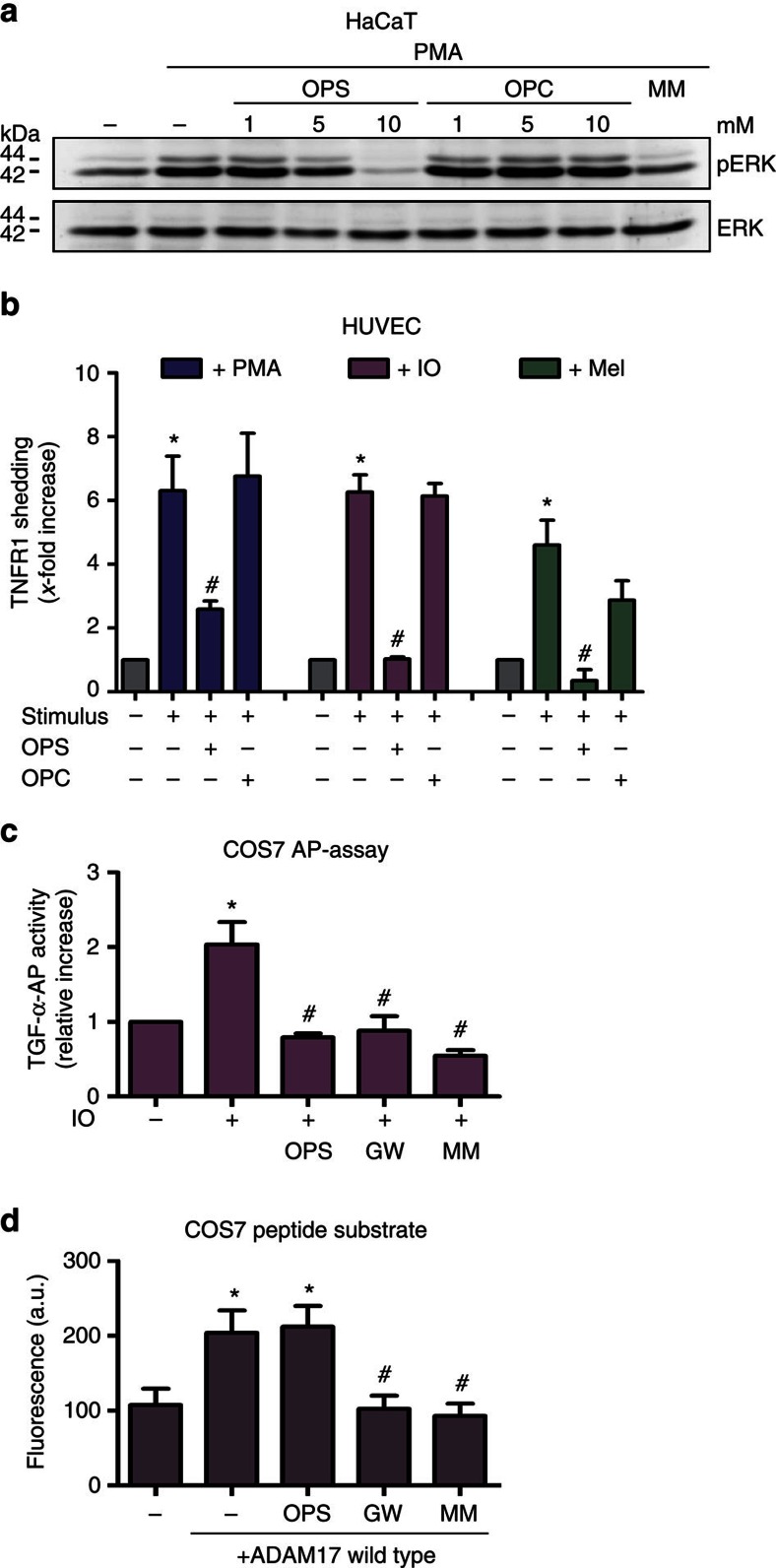
Direct PS interaction is required for ADAM17 activation. (**a**) HaCaT keratinocytes were stimulated with PMA (300 ng ml^−1^) for 30 min and analysed for ADAM17-dependent ERK1/2 activation by western blotting. PMA-induced ERK1/2 phosphorylation was dose dependently reduced by addition of the competing phosphatidylserine head group (OPS) but not by the head group of PC (OPC). Representative western blotting of three independent experiments. (**b**) HUVECs were stimulated with IO (1 μM, 30 min), PMA (200 ng ml^−1^, 60 min) or melittin (Mel, 1 μM, 60 min) in the presence of OPS (10 mM) or OPC (10 mM) and analysed for the release of soluble TNFR1 by enzyme-linked immunosorbent assay (ELISA) (*n*=5; ±s.e.m). (**c**) COS7 cells transfected with AP-tagged TGF-α were analysed for IO-induced (1 μM, 30 min) shedding in the presence of OPS (10 mM), metalloprotease inhibitor MM (10 μM) or ADAM17/10 inhibitor (GW, 3 μM) (*n*=3; ±s.e.m). (**d**) COS7 cells were transfected with ADAM17 and protease cell surface activity was determined by addition of a soluble fluorogenic ADAM peptide substrate. Cell surface activity of ADAM17 was abolished in the presence of GW and MM, but not in the presence of OPS (n=3; ±s.e.m). *A significant increase compared with unstimulated cells (*P*<0.05). ^#^Significant decrease compared with stimulated cells (*P*<0.05). Data were analysed by one-way analysis of variance and Bonferroni multiple comparison *post hoc* test.

ERK1/2 phosphorylation in HaCaT keratinocytes in response to PMA stimulation was dose dependently inhibited by OPS, but not by the PC head group O-phosphocholine (OPC) (Fig. 6a). Release of TNFR1 can be induced in human umbilical vein endothelial cells (HUVECs) by diverse stimuli. Inhibitor experiments confirmed that ADAM17 is significantly involved in this process (Supplementary Fig. 7). Stimulation of HUVECs in the presence of 10 mM OPS or OPC yielded results comparable to the previous data. OPS but not OPC suppressed shedding of the TNFR1 (Fig. 6b). A similar picture emerged for the release of AP-tagged TGF-α from transfected COS7 cells (Fig. 6c).

Fluorogenic peptide substrate assays were then performed in an attempt to clarify whether OPS might directly affect the enzyme. COS7 cells can be transfected to an extent that permits surface-expressed ADAM17 activity to be detected under cell culture conditions. Of note, stimulation of COS7 cells with PMA led to no increase in peptide turnover (Supplementary Fig. 8). Thus, *bona fide* peptidolytic activity and substrate cleavage are distinguishable entities.

Cleavage of the fluorogenic peptide substrate was inhibited by the broad-spectrum metalloprotease inhibitor MM and the inhibitor GW. Conspicuously, however, no inhibitory effect of OPS on cleavage of the soluble substrate was observed. It followed that OPS was not directly interfering with the catalytic function of the protease (Fig. 6d).

### The flexible MPD of ADAM17 binds to PS

**Figure 7:**
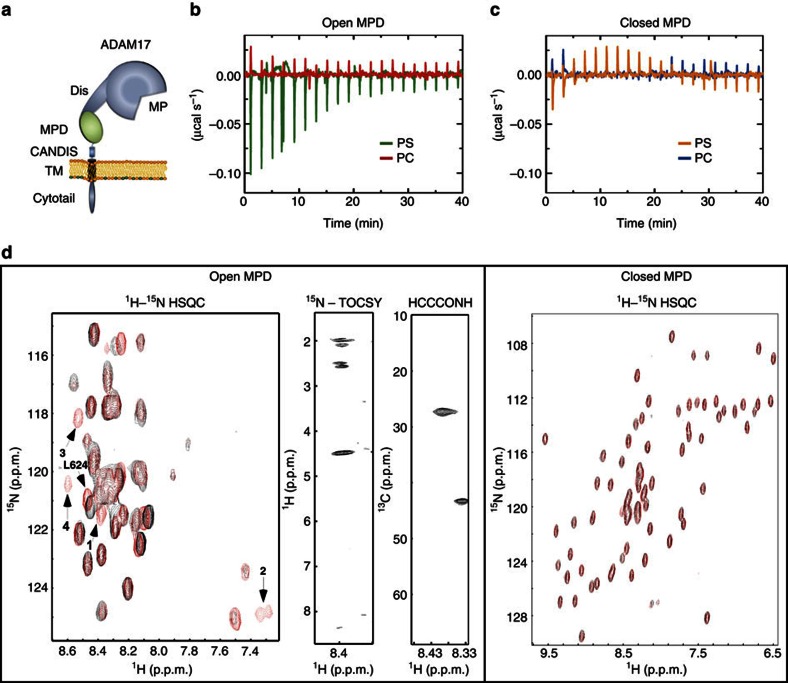
The MPD of ADAM17 binds PS. (**a**) Scheme of ADAM17 structure: metalloprotease domain (MP), disintegrin domain (DIS), MPD, conserved ADAM17 dynamic interaction sequence (CANDIS), transmembrane region (TM) and cytotail. (**b**) ITC measurements to study the interaction of the open MPD with either PS (green) or PC (red) liposomes. (**c**) ITC measurements of the PDI-treated ‘closed' MPD. The MPD was titrated 20 times into a solution containing either PS (orange) or PC (blue) liposomes. No reaction could be observed. (**d**) NMR analysis of the open MPD (left panel) and closed MPD (right panel)–OPS interaction. Overlay of a close-up of the ^1^H-^15^N-HSQC spectra of the MPD conformation in the absence (black) and in the presence (red) of OPS. All resonances of the open conformation (without OPS) appear also in the spectra in the presence of OPS. Four new resonances (arrows) were observed, indicating that the amino acid residues responsible for the OPS binding are located in the flexible part of the MPD. The PDI-treated MPD did not show any reaction in the presence of OPS (right panel).

The collective findings indicated that the release of transmembrane substrates relied on physical interaction of ADAM17 with surface-exposed PS. It appeared likely to be that the site for such an interaction would be located close to the membrane and the MPD of ADAM17 (Fig. 7a) presented itself as the most probable candidate. Recombinant MPD was prepared and employed in experiments using isothermal titration calorimetry (ITC). In contrast to the control (Supplementary Fig. 9), thermic reactions indicative of protein–lipid interaction were indeed detected when the MPD was titrated into buffer solutions containing PS but not PC liposomes (Fig. 7b).

Protein-disulfide isomerase (PDI) can induce a conformational change in the isolated MPD. The carboxy-terminal part of the non-PDI-treated MPD, also referred to as ‘open' MPD, is largely unstructured and flexible, but becomes structured and inflexible due to isomerase-mediated rearrangement of disulfide bonding^42^. When the PDI-treated MPD was injected into a solution containing PS liposomes or PC liposomes in ITC experiments, no reaction could be observed (Fig. 7c), indicating that the PS-binding motif must be part of the PDI-affected flexible region (Supplementary Fig. 10). NMR spectroscopy was used to further examine the binding of OPS to both MPD forms. ^1^H-^15^N-HSQC spectra revealed a specific interaction when OPS was added to the flexible open MPD (Fig. 7d, left panel) but not on addition to the PDI-treated MPD (Fig. 7d, right panel).

Three-dimensional heteronuclear NMR experiments were performed, to identify the PS-interacting amino acid side chains. Resonance 1 could be identified as a lysine residue proceeded by a glycine, whereas resonance 2 was indicative of the NH resonances of an arginine side chain. The resonance of L624 was slightly shifted on the addition of OPS, indicating close proximity to the OPS-binding site. The MPD sequence contains one eligible motif, namely R625/K626/G627/K628 (Fig. 7d, left panel).

### A cluster of basic amino acids mediates MPD–PS interaction

Plasma coagulation factors and lactadherin bind with high affinity to surface-exposed PS either directly via discoidin-like domains or indirectly via Ca^2+^ bridges^43^, but none of the responsible motifs are present in the MPD. Interactions of cytosolic proteins such as Src kinase with ionic phospholipids are, in contrast, typically governed by electrostatic attraction of clusters of cationic amino acids to the membrane^44^ and the possibility arose that such a situation might exist for ADAM17.

**Figure 8:**
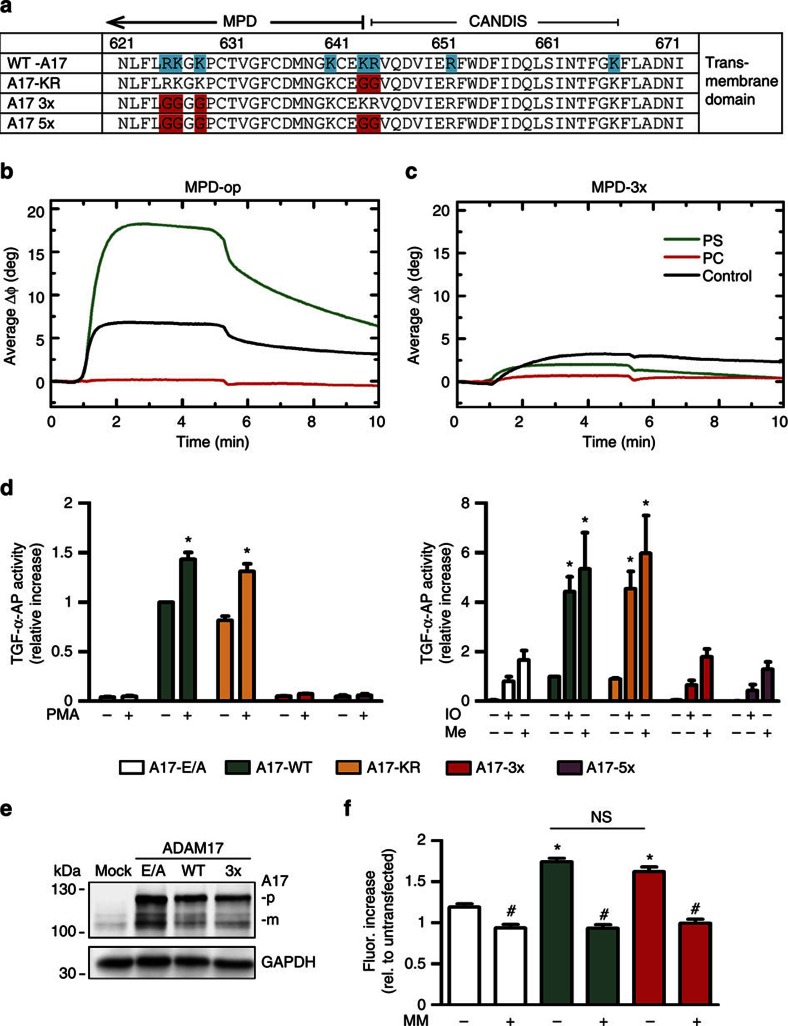
Deletion of the PS-binding motif prevents MPD–PS interaction. (**a**) Potential PS-binding motifs were exchanged creating MPD and ADAM17 mutants: MPD/ADAM17-3x (R625G/K626G/K628G), ADAM17-KR (K643G/R644G) and the combination of both (ADAM17-5x). (**b**,**c**) Binding of the open MPD to CM-dextran/poly-L-lysine (PLL) functionalized sensor chip surface (black) and to immobilized PS membranes (green) or to PC:PS (9:1) membranes (red) in SAW biosensor experiments. Shown is the average phase shift ΔΦ of five individual sensor channels, indicating a mass loading on the surface. (**c**) Mutation of the RK_K motif in the MPD (MPD-3x) abrogates PS binding. (**d**) ADAM17/ADAM10 double-deficient MEFs were cotransfected with AP-tagged TGF-α (TGF-α-AP) and WT-ADAM17, inactive ADAM17 (A17 E/A) or ADAM17 mutants and either stimulated with PMA (200 ng ml^−1^) for 2 h (left panel) or with IO (1 μM) and melittin (Mel, 4 μM) for 30 min (right panel). AP activity in the supernatant was calculated in relation to total (supernatant and cell pellet) AP activity (*n*=3; ±s.e.m.). *A significant increase compared with unstimulated cells (*P*<0.05). (**e**) Transfection efficiency was controlled in parallel by western blot analyses (p=pro, m=mature ADAM17). (**f**) Overexpression of ADAM17-WT or the ADAM17-3x mutant in COS7 cells led to similar significant increases in *bona fide* peptidolytic activity in soluble peptide substrate assays (*n*=3;±s.e.m.; *P<0.05). Metalloprotease inhibitor MM significantly reduced this activity (*n*=3; ±s.e.m.; ^#^*P*<0.05). NS, nonsignificant. Data were analysed by one-way analysis of variance and Bonferroni multiple comparison *post hoc* test.

Recombinant MPD was produced in which the three basic amino acids R625/K626/K628 were mutated to neutral glycins (MPD-3x) (Fig. 8a). Intact protein mass spectrometry (MS) confirmed that all ten cysteines were involved in disulfide linkages (Supplementary Fig. 11a) and liquid chromatography–tandem MS (LC–MS/MS) experiments performed on peptides formed by proteolysis confirmed the presence of a disulfide-linked key peptide proving the ‘open' conformation of this mutant MPD^42^ (Supplementary Fig. 11b).

Non-mutated and MPD-3x were employed in surface acoustic wave experiments using biosensor chips coated with either PC or PS liposomes. Non-mutated MPD was found to bind to PS but not to PC liposomes (Fig. 8b). Despite the presence of several remaining positively charged residues, this PS-binding capacity was completely abolished in the open MPD-3x mutant (Fig. 8c).

To assess the relevance of this motif and neighbouring positively charged amino acids for sheddase function, ADAM17 mutants were constructed in which glycine replaced either K643/R644 (A17-KR) or R625, K626 and K628 (A17-3x) or both motifs (ADAM17-5x). Sheddase function was assessed in murine embryonic fibroblasts that were co-transfected with AP-tagged TGF-α. Although ADAM17 is the major sheddase of TGF-α under normal conditions, ADAM10 releases this substrate in the absence of ADAM17 (ref. 7). Therefore, ADAM10/ADAM17 double-deficient cells were employed for retransfection experiments. These cells were derived from the corresponding knockout embryos that die around embryonic day 8.5 and show severe developmental defects quite similar to ADAM10 single deficient mice^45^ (Supplementary Fig. 12).

As shown in Fig. 8d, shedding of TGF-α was induced by PMA, IO and melittin in cells transfected with wild-type (WT)-ADAM17 but not with the enzymatically inactive ADAM17-E/A mutant. Transfection with the ADAM17-KR mutant also completely restored shedding activity. However, shedding was almost completely absent in cells re-transfected with ADAM17-3x. This effect was not further influenced by additional deletion of the KR-motif (ADAM17-5x).

Exit from the endoplasmic reticulum and maturation of ADAM17 are dependent on interaction with proteolytically inactive members of the Rhomboid family of intramembrane serine proteinases, iRhom1 and iRhom2 (refs 18, 19), and introduction of mutations might affect these processes. However, western blot analyses disclosed that lack of sheddase activity was not due to differences in protein expression or maturation of the ADAM17-3x mutant compared with WT-ADAM17 (Fig. 8e). Flow cytometric analysis revealed comparable cell surface expression of both constructs (Supplementary Fig. 13). These findings were corroborated by fluorogenic peptide substrate assays in COS7 cells demonstrating comparable cell surface-associated peptidolytic activity (Fig. 8f). In particular, these data showed that shedding-incompetent ADAM17-3x retained its enzymatic activity.

## Discussion

**Figure 9:**
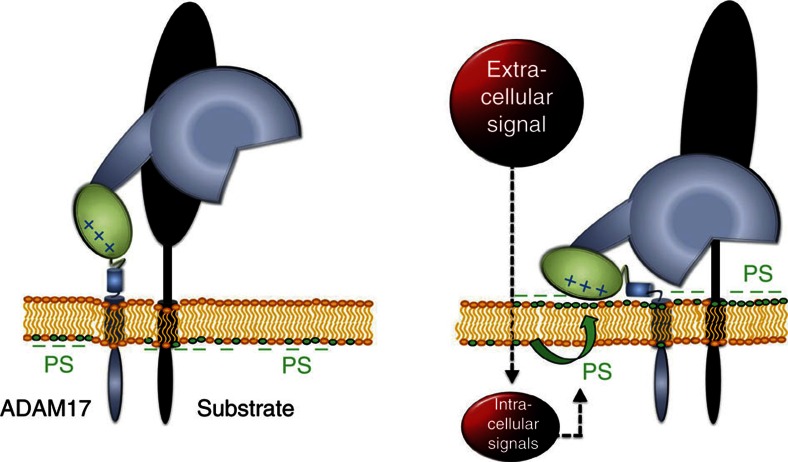
Model of ADAM17 membrane interaction. External stimuli activate intracellular signalling cascades leading to stimuli and cell-type-specific induction of PS externalization from the inner to the outer cell membrane leaflet. Cationic amino acid residues in the MPD of ADAM17 interact with the negatively charged PS head group. The amphipathic α-helical CANDIS could contribute to cell membrane binding, bringing the protease into position for substrate processing.

Remarkably diverse agents rapidly trigger ADAM17-mediated shedding of its substrates from the cell surface. No model exists to explain this perplexing phenomenon. Here we depart from the conventional view that enhanced sheddase function necessarily derives from a genuine increase in enzymatic activity. Instead, we propose that a final event common to and essential for all pathways is surface exposure of PS, which relays intracellular signalling events to the ectodomain of ADAM17. Cationic amino acid residues in the MPD then interact with the negatively charged PS-head group, bringing the protease into position for substrate processing (Fig. 9).

At the cytosolic face, PS subserves essential docking functions for enzymes such as Src kinase, MARCKs and PKC, which interact via clusters of cationic amino acids with the negatively charged phospholipid head group^43^,^46^. In a related manner, the accessory HIV-1 protein NEF associates with membranes in a biphasic process. A fast protein–lipid interaction driven by electrostatic attraction of a basic amino acid cluster (R_R_RR) to PS is followed by formation of an amphipathic helix that subsequently also becomes membrane bound^47^.

In the present study, the cationic cluster RK_K located in the MPD of ADAM17 was found to mediate its interaction with and binding to PS-containing membranes. Intriguingly, the ADAM17 stalk region CANDIS (conserved ADAM17 dynamic interaction sequence)^48^, which follows the MPD onward to the bilayer has recently been found to represent a membrane-interacting amphipathic helix that is also required for sheddase function^49^. The possible similarities in the mode of membrane interaction between HIV-1 NEF and ADAM17 are striking.

A concept emerges in which ADAM17 is envisaged to basically assume two orientations at the cell surface. In the quiescent state, MPD and CANDIS region are freely oriented in the pericellular space and the protease remains poised at a distance from its targets. Binding of the MPD and CANDIS to the lipid bilayer re-orients the protein, drawing its catalytic domain down to its substrate. Simple spatial considerations then explain why substrate cleavage by ADAM17 regularly occurs close to the membrane surface and on the same cell^12^. To date, ADAM17-mediated shedding has indeed never been observed in *trans*.

The proposed model of ADAM17-PS interaction satisfactorily resolves another puzzle relating to the fact that systematic overexpression of the protease does not lead to enhanced shedding activity *in vivo*^50^. Should our idea be correct, shedding will be dependent not simply on levels of ADAM17 expression but also on the dynamics of PS exposure. ADAM17 overexpression alone can thus not be simply equated with increased shedding function.

Our study does not address events that regulate maturation and trafficking of ADAM17, but focuses solely on the possible significance of PS exposure for activity of the sheddase after its positioning at the membrane surface. There, translocation of the anionic phospholipid from the inner to outer leaflet is proposed to conclude events leading to execution of shedding function. PS exposure may occur at different sites and with differing kinetics dependent on the cell type and the stimuli, and surface-exposed PS may then act in concert with intracellular events to control the action of the protease or the accessibility of the substrate. In this context, Dang *et al*.^51^ have presented evidence that PKC-dependent phosphorylation guides lateral movement of the protease to its substrates in the membrane. Furthermore, Maretzky *et al*.^52^ have found that interaction of iRhom1/2 with ADAM17 is sometimes required for triggering sheddase function. Perhaps such processes are responsible for substrate selection and are concluded by PS translocation at the respective sites.

Transient PS exposure in the absence of apoptosis has been described particularly for activated immune cells. Chemotactic activation of neutrophils leads to cell polarization and to PS exposure at the uropod^53^, where ADAM17 and L-selectin have also been shown to co-localize in stimulated cells^54^. It is tempting to speculate that the stimulus-dependent variation in PS exposure we have observed (Fig. 1) bears significance for the orchestration of sheddase function in general. This open question notwithstanding the idea that substrate selection rests on separate but coordinated events at the outside and inside of the cells obviates the need to postulate the existence of a single regulatory element. With regard to a possible role of PS in also attracting ADAM17 substrates, their large number and diversity render it a priori unlikely that all should have membrane-proximal PS-interacting motifs. Indeed, candidate clusters of cationic amino acid residues are not present in TGF-α.

Transiently occurring PS translocation may subserve a number of previously unsuspected functions in living cells. Pathways leading to PS exposure are intricate and often have not been clearly delineated. Several families of scramblases have been identified that cause breakdown of membrane phospholipid asymmetry in nucleated cells. Fortuitously, cells carrying a mutated TMEM16F scramblase from a patient with Scott syndrome were available and provided a valuable tool in the present study. TMEM16F scramblase is activated by intracellular Ca^2+^. This response is selectively defective in Scott syndrome, while mechanisms for PS externalization provoked via other pathways remain intact. Strikingly, IO simultaneously failed to provoke PS exposure and L-selectin shedding in Scott lymphocytes. In contrast, both events occurred on induction of apoptosis.

Use of Raji cells demonstrated that the apoptotic process itself cannot activate sheddase activity when there is no PS exposure.

Experiments with PSA-3 cells that are compromised in their PS synthesizing capacity accorded nicely with these findings. The PS content of these cells could be normalized simply by culture in the presence of ethanolamine. It thus became possible to quite specifically study the influence of PS on sheddase function. Satisfyingly, cells with reduced PS exposed little PS and this was associated with blunted ADAM17 sheddase activity that returned to normal on PS repletion.

The fact that the close correlation between PS exposure and ADAM17 activation observed with the different stimuli and occurring in different cells should represent mere coincidence appeared highly unlikely. Evidence for an interplay between PS and the protease was subsequently obtained in a series of experiments. First, it was found that addition of soluble OPS but not of OPC to cells countered the shedding process. This occurred in the absence of any direct effect of OPS on peptidolytic activity, as was shown by unaltered cleavage of a soluble peptide substrate. It followed that soluble OPS most probably competed for interaction of the protease with the membrane-exposed phospholipid. The propensity of MPD to bind PS was then decovered and the responsible motif identified. On removal of the PS-binding motif, the mutated protease continued to be expressed in its mature form and exposure at the cell surface was ascertained by flow cytometry and through assessment of cell-associated peptidolytic activity using the fluorogenic peptide substrate. The shedding experiments were conducted with ADAM10/ADAM17 double-deficient cells to guarantee that any measured sheddase activity derived solely from the transfected enzyme. Despite its ability to cleave the peptide substrate, mutant ADAM17 lacking the PS-binding motif was unable to shed TGF-α.

Further work with a panel of mutants is needed to obtain deeper insight into the events that govern the interaction of ADAM17 with the membrane and with its substrates. It is noteworthy that the amount of PS residing in the extracellular membrane leaflet can vary considerably depending on cell population, differentiation and activation status^55^,^56^. It will naturally be of interest to investigate whether any of the present findings extend to ADAM10, the second prominent member of the sheddase family. Regulatory mechanisms that govern the function of this closest homologue to ADAM17 have remained equally elusive in the past.

A critical question is whether the present concept would stand in conflict with previous studies that have presented evidence for increased enzymatic activity of ADAM17 in PMA-treated cells. Perhaps, surprisingly, closer inspection of the published data reveals that they actually accord excellently with our model. PMA-mediated increase in cellular cleavage of peptide substrate reported by Doedens *et al*.^26^ rose from basal constitutive levels of 2–3% to only 5–6% in stimulated cells within 60 min. The reason for this low increase in net peptide cleavage was revealed in the recent study of Wu *et al*.^57^. In elegant single-cell measurements, the authors demonstrated that stimulation of HepG2 cells with 100–200 nM PMA resulted in elevated cell-associated protease activity in <5% of the cells over a time span of 36 min. These numbers rose at higher concentrations of the stimulus but still did not exceed 25% at the overtly cytotoxic dose of 1 μM PMA. Given that substantially lower concentrations provoke rapid and maximal substrate shedding of ADAM17 substrates in living cells within minutes^4^,^8^, it follows that in the vast majority of cells the shedding process is not associated with or dependent on any genuine increase in protease activity. This conclusion is supported by our present finding that ADAM17-transfected COS7 cells display no alteration in surface-associated protease activity in response to prolonged stimulation with PMA.

A final conceptual step is now taken. We propose that *bona fide* ADAM17 enzyme activity and sheddase function need to be regarded as separate entities. A mechanistic explanation is provided that assigns a cardinal role of membrane asymmetry in controlling the function of the extracellularly oriented membrane protein. PS exposure can be expected to occur at differing sites and vary in duration and intensity depending on the trigger, cell type and local scramblase machinery. A unique stage could thus be set for the central sheddase of the cell to assume its multifaceted roles in biology.

## Methods

### Reagents and antibodies

Annexin V–FITC, phosphorylserine (OPS), phosphocholine (OPC), PMA, staurosporine, suramin and IO were obtained from Sigma. PS and PC were from Avanti Polar Lipids. Hydroxamate-based ADAM17/ADAM10 inhibitor GW280264 (ref. 58) was purchased from Aeobious. MM and ADAM10 inhibitor GI254023X were purchased from Tocris Bioscience. pSIVA IANBD was from Imgenex Corp. PP2 was from Calbiochem. Z-VAD-FMK and anti-GAPDH (sc-25778, 1:500) were from Santa Cruz Biotechnology. POD-coupled secondary antibodies for immunoblot were from Dianova (705-035-147, 1:10,000). Melittin was synthesized as described^59^. ‘Open' and ‘closed' MPD was produced and prepared as described^42^. The neutralizing ADAM17 antibody D1 (A12) was purchased from AdipoGene Life Sciences (AG-27B-6000PF-C100). Anti-tubulin (E7, 1:500) was from DSHB. Anti-phosphoERK1/2 (4370) and anti-ERK1/2 (4695) were from Cell Signalling (1:3,000). EGFR-blocking antibody Cetuximab (C225) and anti-Fas Antibody CH11 (05-201) were from Merck Millipore. Anti-ADAM17 antibodies used for western blotting (clone 10.1, 1:1,000) and flow cytometric analysis (K133) were described previously^60^. Additional antibodies used for flow cytometry were as follows: L-selectin (BD Bioscience, DREG56, 559,772, 1:100) and IgG1-APC (BD Bioscience, 554,681, 1:100), rabbit IgG1 control (Southern Biotechnology, 0111-01, 1:100), anti-rabbit Alexa Fluor 488 (Life Technologies, R37118, 1:100) and anti-TNFR1-APC (R&D, FAB225A, 1:100).

### Cell culture

HaCaT cells were provided by Dr N.E. Fusenig (DKFZ, Heidelberg, Germany)^61^. Mouse embryonic fibroblasts (MEFs) from ADAM10/17^−/−^ mice were generated as described below. Human Jurkat T cells and Epstein-Barr virus (EBV)-transformed Raji B cells were purchased from the American Type Tissue Collection (ATCC). MEFs, HaCaT and COS7 cells (ATCC) were grown in high-glucose DMEM (Thermo Fisher Scientific) supplemented with 10% FCS and 1% penicillin/streptomycin (Pen/Strep). HUVEC cells (Provitro) were cultured in endothelial cell growth medium (PromoCell). Epstein-Barr virus (EBV)-transformed B-lymphoblast cell lines from control subjects and a Scott UK patient have been described before^62^,^63^. Lymphocytes were grown in RPMI-1640 medium (GIBCO) supplemented with 10% FCS (GIBCO) and Pen/Strep. PSA-3 cells were cultivated in Ham's F12 medium supplemented with 10% FCS, 10 μM ethanolamine (Sigma) and 1% Pen/Strep. For PS deprivation, cells were cultured in starvation medium consisting of Hams F12 with 6% dialysed FCS+Pen/Strep with or without 10 μM ethanolamine^64^.

### Expression vectors

The expression vectors for murine WT-ADAM17, inactive ADAM17-E/A mutant were from Dr Gillian Murphy (Cambridge, UK). The plasmid for AP-tagged TGF-α expression was from Dr Carl P. Blobel (Hospital for Special Surgery, New York, USA).

### Generation of ADAM17 mutants

Quikchange II site-directed mutagenesis kit (Agilent Technologies) was used according to the manufacturer's instructions. The mutations included ADAM17-3x (R625G, K626G and K628G substitutions), ADAM17-KR (K643G and R644G substitutions) and ADAM17-5x (R625G, K626G, K628G, K643G and R644G substitutions). The primer sequences for the ADAM17-3x mutations were 5′- gcagagca aaagaacttgtttttggggggaggggggccatgtacagta gggttttgcg -3′(fwd), 5′- cgc aaaaccctactgtacatggcccccctccccccaaaaacaagttcttttgctctgc -3′(rev) and ADAM17-KR mutations were 5′- gacatgaatggcaaatgtgagggag gagtacaggacgtaattgagc -3′(fwd), 5′- gctcaattacgtcctgtactcctccctcac atttgccattcatgtc -3′(rev). Successful mutation was verified by sequencing (Seqlab).

### Generation of MPD-3x

Cloning, bacterial expression and purification of the MPD-3x was performed by Trenzmye GmbH. The complementary DNA of human MPD (amino acid residues F581–E642 with the following changes: R625G/K626G/K628G) was cloned into pTZ_E03 expression vector and transformed into *Escherichia coli* strain Rosetta 2 (DE3). The soluble expressed domain was purified in PBS by its amino-terminal His tag using nickel affinity chromatography.

### Transfection and AP-substrate shedding assay

ADAM10/ADAM7 double-deficent MEFs were transfected using Turbofect Transfection Reagent (Thermo Fisher Scientific) according to the manufacturer's instructions. Twenty-four hours after transfection, cells were washed with DMEM, which was replaced 1 h later by fresh DMEM and treated or not treated with PMA (200 ng ml^−1^, 2 h) or IO (1 μM, 30 min), or melittin (4 μM, 30 min). Thereafter, supernatants and cell lysates were collected and measured for AP activity at A405 employing the AP substrate 4-nitrophenyl phosphate (Sigma-Aldrich). Shown is the relative AP activity in the supernatant compared with the total AP activity of supernatant plus cell lysates normalized to the unstimulated A17-WT. For shedding assays, the cells were lysed in buffer containing 2.5% Triton-X, 10 mM 1.10-phenanthroline and 1 mM EDTA in water. The metalloprotease inhibitor phenanthroline inhibited autocatalysis of ADAM17 (ref. 65). The expression level of ADAM17 constructs was analysed in parallel by western blot analyses 24 h after transfection.

For AP assay experiments in PSA-3 cells, cells were transfected after 72 h PS deprivation for 6 h in Ham's F12 without FCS. Thereafter, medium was changed again to the starvation medium. All AP assays and imaging experiments were performed after 96 h of PS deprivation. As starvation reduced the transfection efficiency, the stimulation effect was normalized to the non-treated cells, respectively.

### Confocal live-cell imaging

Time-lapse imaging was performed with an inverted confocal microscope (Fluoview FV1000, Olympus) equipped with an environmental chamber using a UPLSAPO × 60 oil-immersion objective (numerical aperture: 1.35) and 2 × zoom. Before starting the experiments the chamber was equilibrated to 37 °C in a humidified atmosphere. The cells were seeded in glass-bottom imaging dishes (PAA) and grown to semi-confluence. Cells were allowed to rest for at least 1 h in the microscope stage. Five minutes before acquisition started, PSIVA-IANBD was added to the cells in a final concentration of 6.25 μg ml^−1^. PSIVA-IANBD was excited at 488 nm and emission was recorded at 520 nm every 30 or 60 s. Laser and detector settings were the same for each single experiment.

### Immunocytochemistry

HaCaTs were seeded on glass coverslips and grown to semi-confluence. After indicated stimulation/inhibition periods, coverslips were immediately incubated with a 1:20 solution of Annexin V–FITC in Annexin binding buffer (ABB) for 5 min in the dark at room temperature, washed twice with ABB (10 mM HEPES, 140 mM NaCl and 2.5 mM CaCl_2_ pH 7.4) and fixed for 15 min with 3% paraformaldehyde. After fixation, coverslips were washed six times with PBS, once with distilled water and mounted in embedding medium. Image acquisition was performed with an inverted confocal microscope (Fluoview FV1000, Olympus) using a UPLSAPO × 60 oil-immersion objective (numerical aperture: 1.35) and 2 × zoom. Annexin V–FITC was excited at 488 nm and emission was recorded at 520 nm. Images were acquired with the same laser and detection settings for each experimental setup, but vary between Figs 1 and 2 because of different Annexin V–FITC lots.

### Image analysis and image statistics

Image analysis was performed with ImageJ 1.47 m. Fluorescence area above background fluorescence was determined and correlated to the cell growth area. Immunocytochemistry for each group, at least three independent coverslips, each with six to eight images of different areas, were analysed. The mean fluorescence area of each coverslip was taken for statistical analysis. Groups were tested by one-way analysis of variance and Bonferroni multiple comparison *post hoc* test.

### Flow cytometric analysis

For analysing ADAM17 cell surface expression, cells were washed with PBS and stained with anti-ADAM17 (K133, 10 μg ml^−1^) or rabbit IgG-control antibodies (1:100) in 100 μl PBS with 3% BSA for 1 h on ice. After washing, cells were incubated with anti-rabbit Alexa Fluor 488 (1:100) for 30 min on ice. Cells were washed and resuspended in PBS.

Kinetic analysis of PS exposure and L-selectin surface expression was performed by staining cells with APC mouse anti-human L-selectin (BD Bioscience) or mouse IgG1 1:100 in 500 μl RPMI with 1% FCS for 30 min on ice. Thereafter, cells were washed twice and resuspended in 190 μl RPMI. Before commencement of the experiments, cells were warmed to 37 °C for 5 min and the media was supplemented with 2 mM CaCl_2_. pSIVA-IANBD (10 μl) was then added and incubated for 3 min. The samples (20 μl each) were diluted in 500 μl ice-cold ABB and analysed by flow cytometry before and after addition of IO (2 μM) at the indicated time points for a period of 6 min.

Staining after Fas-Ab treatment (6 h Scott cells and WT B cells; 2 h Jurkats and Rajis) was performed as follows: cells were stained with Annexin V–FITC (1:100) in ABB for 10 min in the dark at room temperature, washed with ABB and fixed for 15 min with 3% paraformaldehyde on ice. Cells were subsequently washed in PBS and stained for 1 h with the corresponding antibody or control at 4 °C in PBS with 1% BSA (anti-TNFR-APC or isotype control (Jurkats and Rajis), anti-L-selectin or isotype control (Scott cells and WT B cells). After staining, cells were washed and analysed by flow cytometry with FACSVerse (BD Bioscience). Data were analysed using FlowJo 8.7.3.

### Caspase-3 activity assay

Caspase-3 activity was assayed by mixing 50 μl cell lysate (500,000 cells) with 150 μl reaction buffer (100 mM HEPES pH 7.4, 0.1% CHAPS, 10% sucrose, 5 mM dithiothreitol) containing 50 μM of fluorogenic caspase-3 substrate (Ac–DEVD–AMC, Enzo Life Science). The mixture was incubated for 60 min at 37 °C in an enzyme-linked immunosorbent assay plate and fluorescence was measured in the microplate fluorometer Twinkle LB 970 (excitation 355 nm, emission 460 nm). Caspase-3 activity was expressed as factor of fluorescence increase relative to non-treated cells.

### ADAM peptide substrate assay

COS7 cells were transfected in 24-well plates with WT-ADAM17, ADAM17-E/A or ADAM17-3x constructs or with the empty control plasmid (pcDNA3.1). Twenty-four hours after transfection, medium was changed to DMEM with 10 μM fluorogenic ADAM substrate (Enzo Life Sciences) and the cells were incubated for the indicated time points. Fluorescence in the supernatant was determined at 485 nm excitation and 530 nm emission.

### NMR spectroscopy and LC–MS

The ^15^N- and ^13^C-isotope-labelled open and closed conformation of the MPD of ADAM17 was prepared as described previously^42^. NMR measurements were performed on a Bruker Avance 600-MHz spectrometer equipped with a *z*-gradient triple-resonance cryoprobe. The proteins were dissolved in 10 mM phosphate buffer pH 7.4, 0.01% NaN_3_ and 7% D_2_O. OPS was added in a tenfold molar excess. Sequence-specific backbone resonance assignments of the MPD conformers were established using the following spectra: three-dimensional HNCA, HNCO, (H)C(CO)NH, H(CCO)NH, HN(CA)CO, HN(CO)CA, HN(CO)CACB, HNCACB, ^15^N-edited TOCSY and ^13^C-edited HCCH-TOCSY. The spectra were acquired at 300 K and referenced to the water resonance at 4.79 p.p.m. All spectra were processed with NMRPipe^66^ and analysed with NMRViewJ^67^. LC–MS experiments for the determination of MPD-3x disulfide bonds were performed as described before^42^.

### Enzyme-linked immunosorbent assay

TGF-α and TNFR1 enzyme-linked immunosorbent assay (both R&D) were performed according to the manufacturer's instructions. Cells were grown until confluence in 12-well plates. HaCaTs were stimulated with PMA (300 ng ml^−1^), IO (1 μM) or melittin (1 μM) in the presence or absence of MM (10 μM), GI (2 μM) or GW (2 μM) in duplicates for 30 min. HUVECs were stimulated with PMA (200 ng ml^−1^), IO (1 μM) or melittin (1 μM) in the presence or absence of OPS (10 mM) or OPC (10 mM) in duplicates for 1 h. Supernatants were analysed for soluble shedding products in duplicates.

### Western blot analysis

Cells were washed once with PBS and lysed in lysis buffer (5 mM Tris-HCl pH 7.5, 1 mM EGTA, 250 mM saccharose and 1% Triton X-100) supplemented with cOmplete inhibitor cocktail (Roche Applied Science) and 10 mM 1,10-phenanthroline monohydrate to prevent ADAM17 autocleavage^65^. Equal amounts of protein were loaded on 10% SDS–PAGE gels. The samples were electrotransferred onto polyvinylidene difluoride membranes (Hybond-P; Amersham) and blocked overnight with 5% skim milk in Tris-buffered saline. After incubation with the indicated antibody in blocking buffer, the membranes were washed three times in TBST (Tris-buffered saline containing 0.1% Tween-20). Primary antibodies were detected using affinity-purified peroxidase (POD)-conjugated secondary antibodies (1:10,000) for 1 h at room temperature. Detection was carried out using the ECL detection system (Amersham). Signals were recorded by a luminescent image analyser (Fusion FX7 imaging system; PEQLAB Biotechnologie). To analyse the expression of pERK1/2 in comparison with total ERK1/2, western blottings were incubated in stripping reagent (100 mM 2-mercaptoethanol, 2% (w/v) SDS, 62.5 mM Tris-HCl pH 6.7) at 55 °C for 30 min and re-probed with the exception of the control blot in Fig. 6a. Here, protein samples were loaded on separate western blottings and probed as indicated. Uncropped scans of the blottings are shown in Supplementary Fig. 14.

### Preparation of lipid aggregates/liposomes

Aggregates/liposomes were prepared as 1 mM aqueous dispersions of the phospholipids in buffer (5 mM HEPES and 100 mM KCl pH 7.0) as follows. The lipids were dissolved in chloroform to a concentration of 1 mg ml^−1^. The solvent was evaporated under a stream of nitrogen, buffer was added and sonicated with a Branson sonicator (Branson Ultrasonics Corporation) for 1 min (1 ml solution). Subsequently, the preparation was cooled for 30 min at 4 °C and two times heated for 30 min at 60 °C and cooled to 4 °C. Preparations were stored at 4 °C overnight before measurements.

### Acoustic wave biochip analyses

Gold-coated chips (S-sens K5 Biosensor Quartz Chips, SAW Instruments GmbH) were functionalized as described^68^. A functionalized chip was incubated overnight in ethanol and placed into the reader unit of the S-sens K5 readout system (SAW Instruments GmbH). Biomolecular interaction processes on the surface of the sensor chip can result in changes of phase and amplitude of the surface acoustic wave. Changes of these parameters correlate with mass loading. Measurements were performed at a continuous flow of measuring buffer (5 mM HEPES pH 7.4) of 20 μl min^−1^ at 22 °C. By injection of 100 μl of poly-L-lysine (50 μg ml^−1^, Fluka), a positively charged layer was formed on top of the negatively charged CM–dextran matrix. One hundred microlitres of phospholipid liposomes (300 μg ml^−1^) (PS liposomes or PC(9):PS(1) liposomes) were injected and immobilized on the positively ionized surface. The liposomes were dissolved in 150 mM NaCl and 5 mM HEPES pH 7.4, to ensure a homogenous surface coverage. Subsequently, 100 μl of 20 μg ml^−1^ solutions of the open and closed form of MPD in 5 mM HEPES were injected. Changes of phase and amplitude induced by the interaction of MPD with the lipid bilayer were recorded over time.

### Isothermal titration calorimetry

The binding of the MPD with PC or PS liposomes was analysed by microcalorimetric measurements on an ITC200 (GE Healthcare)^69^. Solutions (0.5 mM) of the open and closed form of MPD in 5 mM HEPES were titrated 20 times in 1.5 μl injections to 250 μl 0.1 mM PS or PC liposome dispersions. Final lipid:protein ratio was 5:3 (M:M). The enthalpy changes were recorded over time. The heat of dilution were determined in control experiments by injecting peptide solution into buffer (5 mM HEPES, pH 7.4). Measurements were performed at 37 °C.

### Generation of ADAM10/ADAM17 double-deficient mice and cells

ADAM10-deficent mice have been described^45^. To generate ADAM10/ADAM17 double-deficient mice, heterozygous ADAM10^−/−^ mice were mated with heterozygous ADAM17-deficient mice, which were generated by gene trap as follows. Embryonic stem cell line RST495 with an intronic gene trap insertion between exon 6 and exon 7 of the *Adam17* gene were obtained from BayGenomics. The embryonic stem cells were injected into the blastocoel cavity of C57Bl/6J-derived blastocysts and implanted into pseudopregnant females. Chimeric mice generated from the injections transmitted the targeted allele to their progeny. Genotyping was performed by intron spanning ADAM17 PCR 5′- tcatcgattttataaatacatgggccg -3′(fwd), 5′- accctgcattatcccacgacgtgttcc -3′(rev) and neomycin PCR 5′- gttgtcactgaagcgggaagggactggctg -3′(fwd), 5′- gcgaacagttcggctggcgcgagcccctga -3′(rev) or β-galactosidase PCR (not shown). Heterozygous ADAM17^+/−^ mice were normal and fertile. They were intercrossed to generate homozygous gene-targeted mice. ADAM17-deficient mice showed a comparable phenotype as has been described for mice with deleted ADAM17 Zn^2+^-binding domain^4^ including eye, hair and skin defects (Supplementary Fig. 12), and embryonic lethality. MEFs were derived from dissociated E16 embryos and maintained in DMEM supplemented with FCS and antibiotics. Total RNA from MEFs was reverse transcribed and subjected to PCR amplification for *Adam17* using the primers 5′- gtacgtcgatgcagagcaaa -3′(fwd) and 5′- aaaccagaacagacccaacg -3′(rev). *Actin* was used as control (5′- gacctctatgccaacacagt -3′(fwd) and 5′- agtactttgcgctcaggagga -3′(rev)). Protein deficiency was confirmed by western blotting using anti-ADAM17 ectodomain antibodies (Merck Millipore). Heterozygous ADAM17^+/−^ mice were intercrossed with heterozygous ADAM10^+/−^ mice. ADAM10/17^−/−^ embryos were evaluated at different stages of development. At day 9 of embryogenesis, embryos presented in a Mendelian frequency (not shown), as evaluated by PCR. No viable ADAM10/17^−/−^ embryos were obtained beyond E9. MEFs were derived from dissociated E9 embryos and maintained in DMEM supplemented with FCS and antibiotics. Protein deficiency was confirmed by RT–PCR (not shown) and western blotting. The functional analysis of these cells is described elsewhere^7^,^22^.

### Statistical analysis

All values for the ectodomain shedding assays are expressed as means± s.e.m. The s.e. values indicate the variation between mean values obtained from at least three independent experiments. Statistics were generated using one-way analysis of variance and Bonferroni multiple comparison *post hoc* test. *P*-values<0.05 were considered statistically significant (either indicated with * or #).

## Additional information

**How to cite this article:** Sommer, A. *et al*. Phosphatidylserine exposure is required for ADAM17 sheddase function. *Nat. Commun.* 7:11523 doi: 10.1038/ncomms11523 (2016).

## Supplementary Material

Supplementary Information Supplementary Figures 1-14

Supplementary Movie 1 pSIVA live cell imaging of HaCaT keratinocyte stimulated with melittin. HaCaT keratinocyte were pre-incubated for 15 min before stimulation with melittin (1 μM) in the presence of pSIVA. This movie corresponds to Fig. 1 a.

Supplementary Movie 2 pSIVA live cell imaging of HaCaT keratinocyte stimulated with ionomycin. HaCaT keratinocyte were pre-incubated for 15 min before stimulation with ionomycin (1 μM) in the presence of pSIVA. This movie corresponds to Fig. 1 b.

Supplementary Movie 3 pSIVA live cell imaging of HUVECs stimulated with ionomycin. Human umbilical vein endothelial cells (HUVECs) were pre-incubated for 15 min before stimulation with ionomycin (1 μM) in the presence of pSIVA.

Supplementary Movie 4 pSIVA live cell imaging of PSA3 cells with ethanolamine stimulated with ionomycin. PSA3 cells were pre-incubated for 15 min before stimulation with ionomycin (1 μM) in the presence of pSIVA. This movie corresponds to Fig. 3 a.

Supplementary Movie 5 pSIVA live cell imaging of PSA3 cells without ethanolamine stimulated with ionomycin. PSA3 cells were pre-incubated for 15 min before stimulation with ionomycin (1 μM) in the presence of pSIVA. This movie corresponds to Fig. 3 a.
