# Supplementary material for: Phosphatidylserine exposure is required for ADAM17 sheddase function
Source: Nat Commun. 2016 May 10;7:11523. doi: 10.1038/ncomms11523 (PMC4866515; doi:10.1038/ncomms11523)
Supplement: Supplementary Information — Supplementary Figures 1-14 [file ncomms11523-s1.pdf]

Supplementary Figure 1

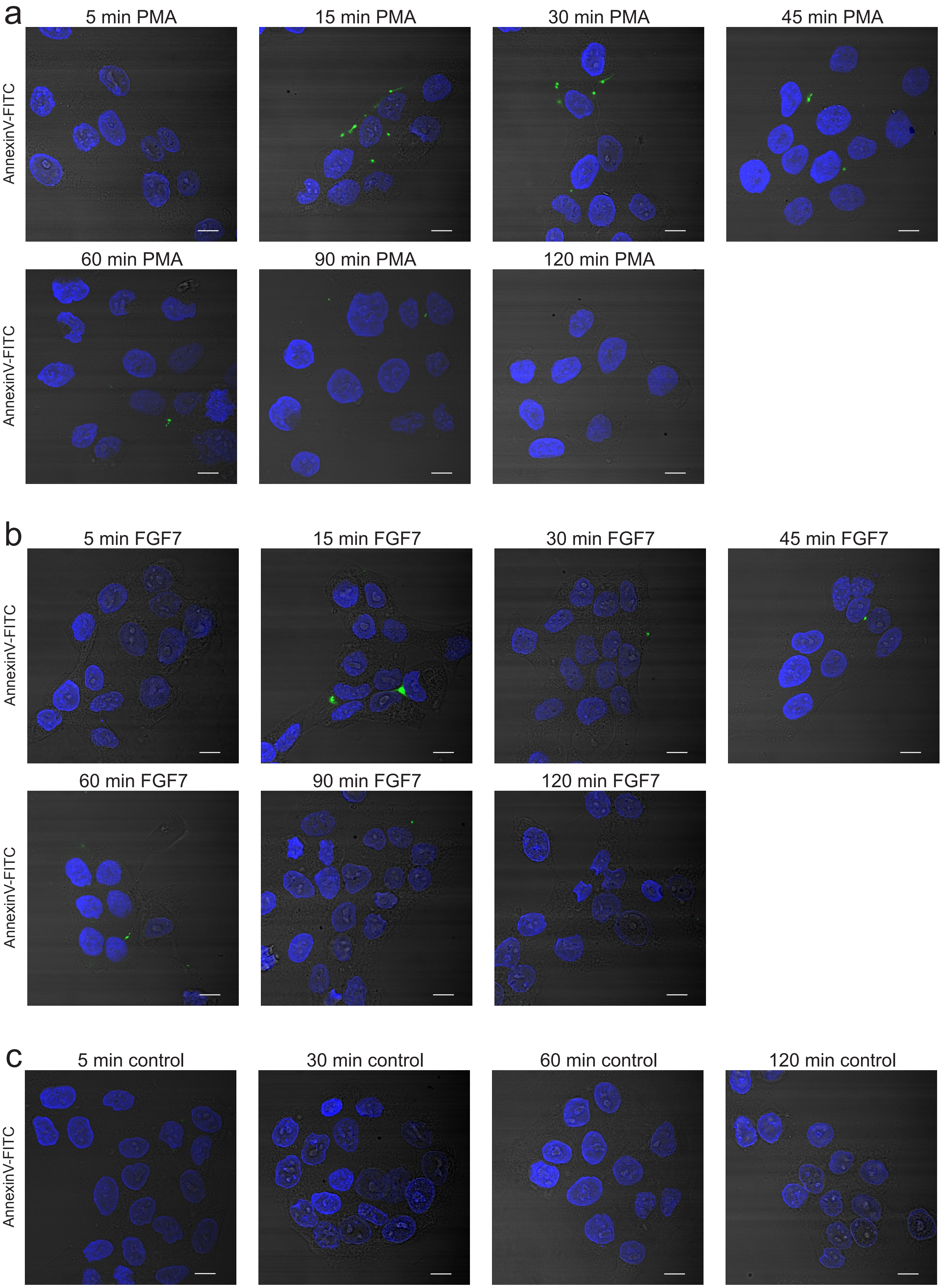

Supplementary Figure 1. PS exposure in HaCaT keratinocytes. Cells were grown on cover slips and stimulated with **(a)** PMA (300 ngml^{-1}) or **(b)** FGF7 (100 ngml^{-1}) or left untreated **(c)** and stained with AnnexinV-FITC after the indicated time points. Representative images of three independent experiments are shown. Scale bars: 10 µm.

Supplementary Figure 2

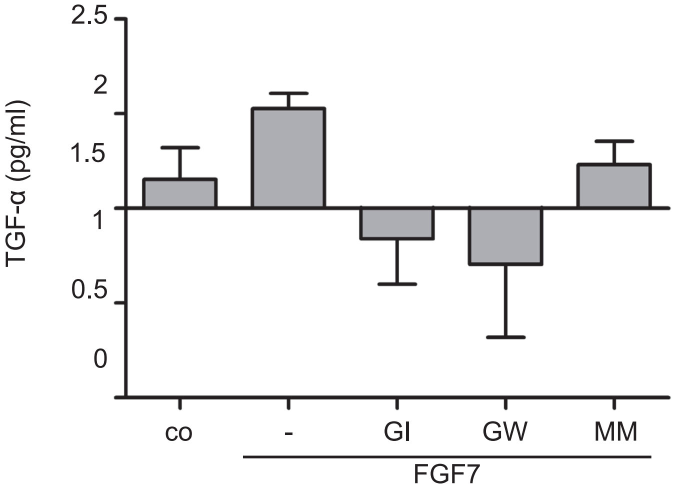

Supplementary Figure 2. FGF7 induces barely detectable quantities of soluble TGF-α. HaCaT keratinocytes were stimulated with FGF7 (100 $ngml^{-1}$) for 30 min. The release of soluble TGF-α was determined in the presence of broad-spectrum metalloprotease inhibitor marimastat (MM, 10 μM), the ADAM17/10 inhibitor GW (3 μM), and the preferential ADAM10 inhibitor GI (3 μM) (*n*=3;± s.e.m).

Supplementary Figure 3

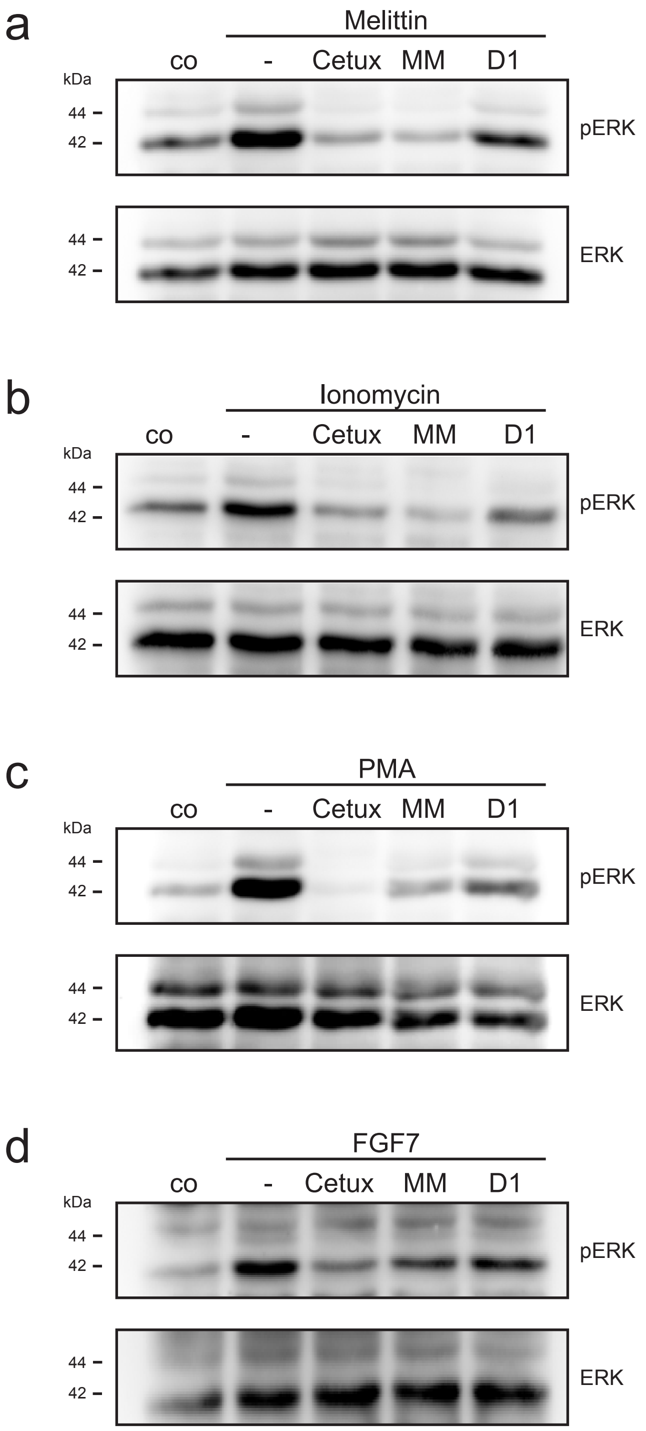

Supplementary Figure 3. Involvement of ADAM17 in ERK1/2 activation in keratinocytes. HaCaT keratinocytes were stimulated with **(a)** melittin (1 µM), **(b)** ionomycin (1 µM), **(c)** PMA (300 $ngml^{-1}$) or ***(d)*** FGF7 (100 $ngml^{-1}$) for 15 min and analysed for ERK1/2 activation in the presence of EGFR-blocking antibody Cetuximab (10 µgml-1), metalloprotease inhibitor marimastat (MM, 10 µM), or ADAM17 blocking antibody D1 (200 nM). Representative western blot analyses of ERK1/2 phosphorylation with an immunoblot of total ERK1/2 included as loading control are shown.

Supplementary Figure 4

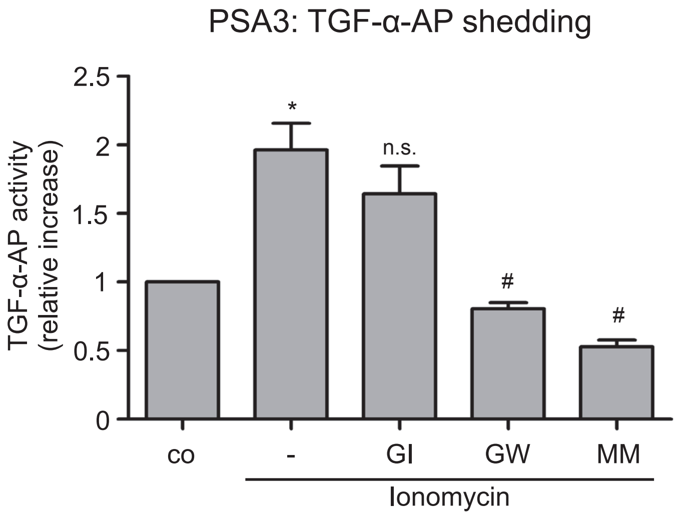

Supplementary Figure 4. TGF-α-AP release in PSA-3 cells. PSA-3 cells were transfected with TGF-α-AP. 48 h after transfection cells were stimulated with ionomycin (IO, 1 μM) and analysed for substrate release. IO treatment significantly increased TGF-α-AP shedding (n=3; ± s.e.m;*P<0.05). The broad-spectrum metalloprotease inhibitor marimastat (MM, 10 μM) and the ADAM17/10 inhibitor GW (2 μM) but not the preferential ADAM10 inhibitor GI (2 μM) significantly reduced the induced proteolysis (n=3;± s.e.m;#P<0.05). N.s.: non-significant.

Supplementary Figure 5

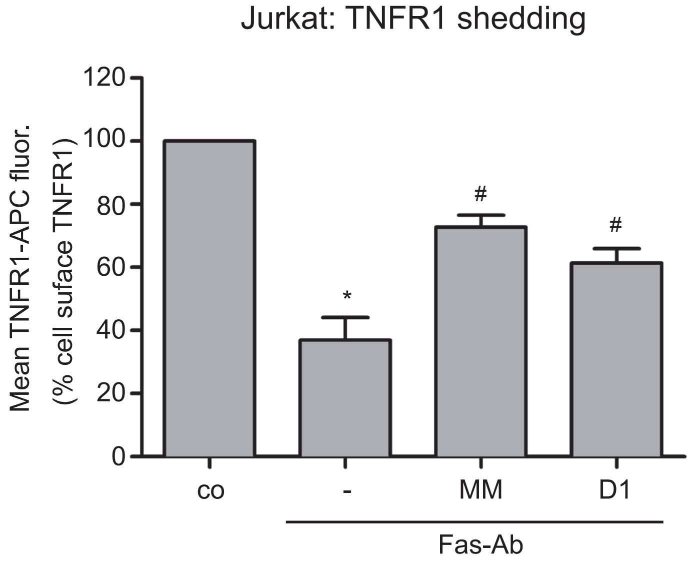

Supplementary Figure 5. Fas-Ab induced shedding of TNFR1 from Jurkat cells. This Figure supplements Figure 4. Jurkat cells were treated with Fas-Ab (500 µgml^{-1}) in the absence or presence of broad-spectrum metalloprotease inhibitor marimastat (MM, 10 µM) or ADAM17 blocking antibody D1 (200 nM) for 2 h. Cells were stained with AnnexinV-FITC and anti-TNFR1 APC-conjugated antibody and analysed by flow cytometry. Mean APC values of Fas-Ab treated AnnexinV-FITC-positive cells were normalized to the mean APC value of AnnexinV-FITC-negative untreated cells (co, 100%) and APC-IgG1 stained control cells (not shown, 0%). Fas-Ab treatment significantly reduced TNFR1 cell surface expression (n=6;± s.e.m;*P<0.05). Co-incubation with marimastat or D1 significantly rescued the loss of TNFR1 in Fas-Ab treated AnnexinV-FITC-positive cells (MM: n=6;± s.e.m; D1: n=3;± s.e.m; #P<0.05).

Supplementary Figure 6

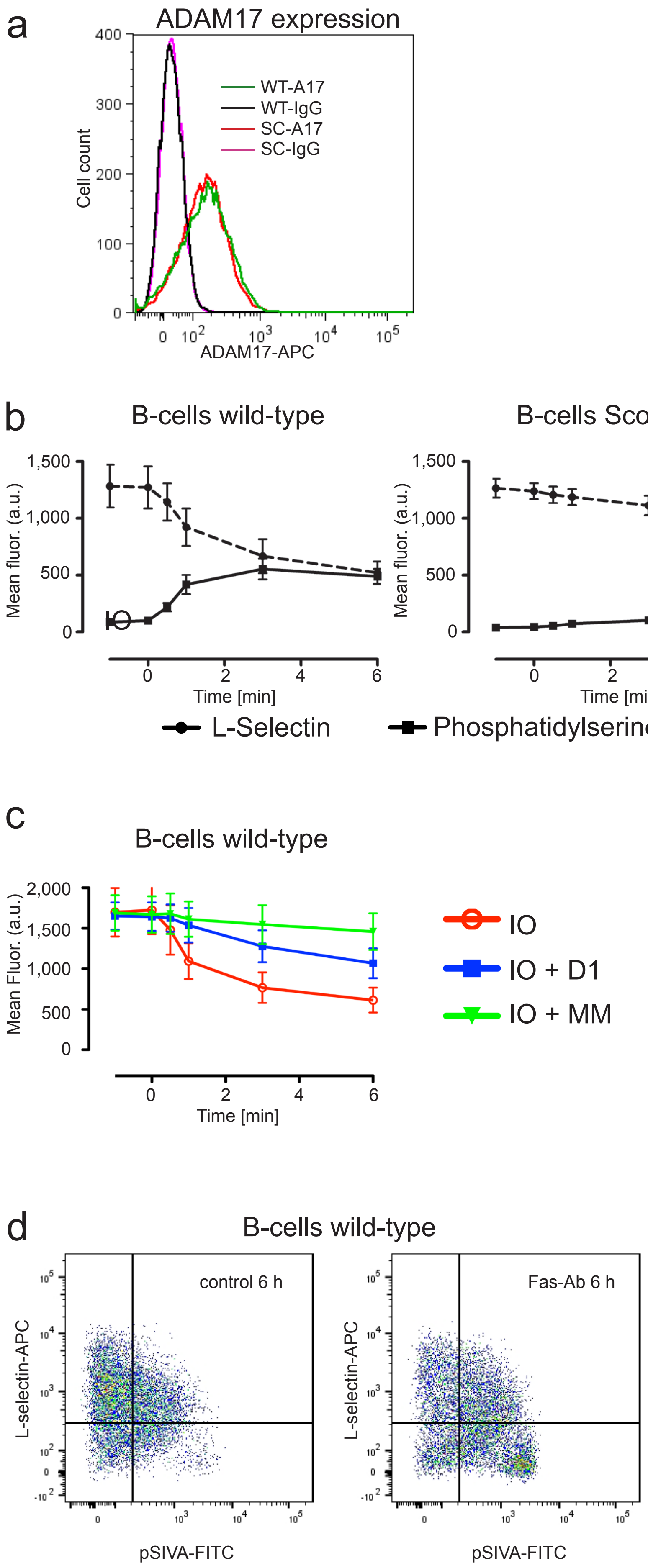

Supplementary Figure 6. Detailed flow cytometric analysis of B-cells. **(a)** The surface expression of ADAM17 vs. isotype (IgG) on B-cells from wild-type (WT) and from Scott patient (SC) was analysed by flow cytometry. **(b)** Time course of surface staining of PS and L-selectin of B-cells wild-type and B-cells from the Scott patient UK after ionomycin (IO; 2 µM) treatment added at 0 min. **(c)** The loss of L-selectin in these cells upon IO stimulation was strongly reduced by marimastat (MM; 10 µM) and the ADAM17-blocking antibody D1 (200 nM) (*n*=3;± s.e.m). **(d)** Apoptosis was induced in wild-type B-cells by Fas antibody (Fas-Ab, 500 ngml^{-1}) treatment for 6 h and surface L-selectin and PS was analysed by flow cytometric analysis.

Supplementary Figure 7

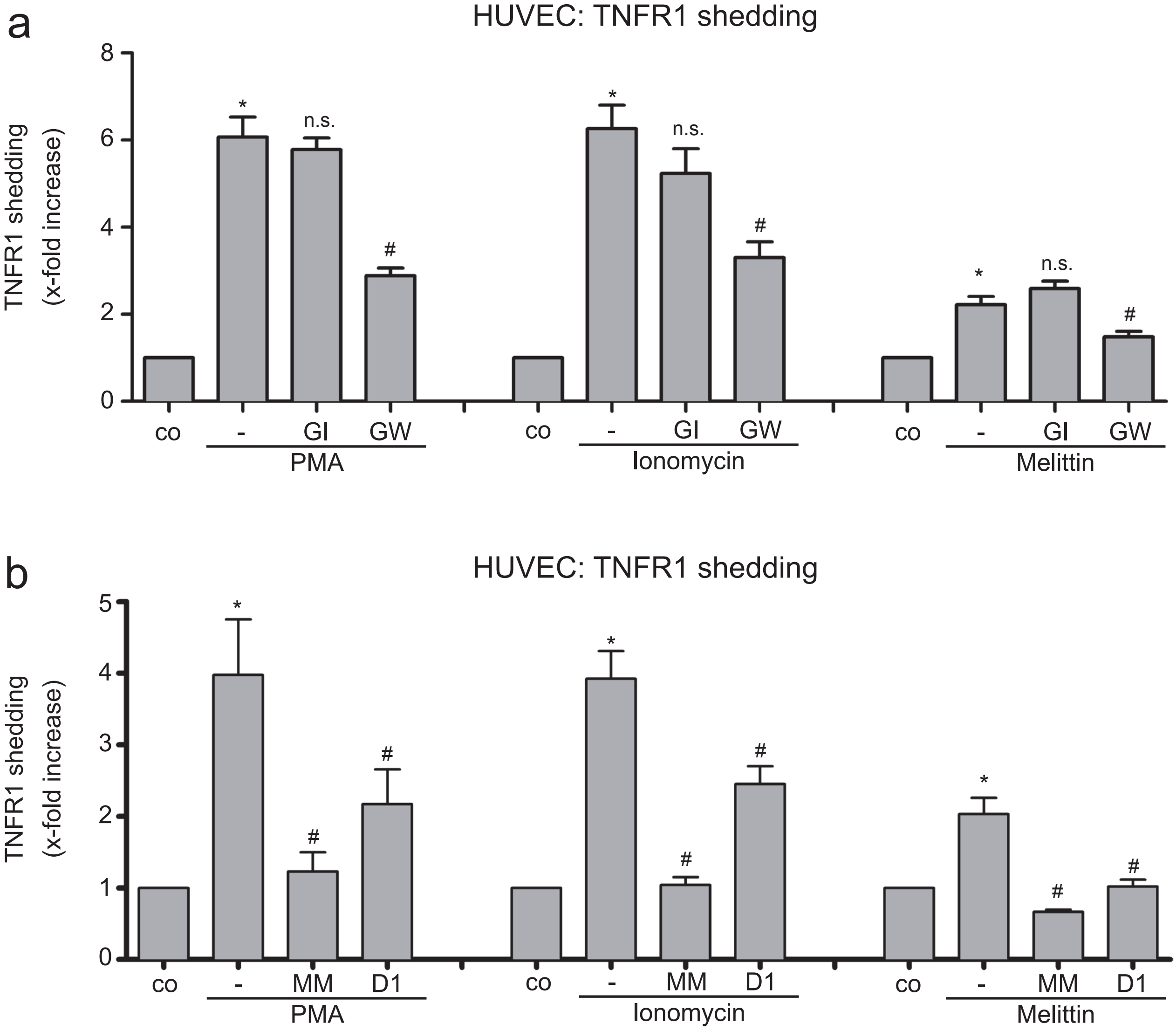

Supplementary Figure 7. TNFR1 shedding in HUVECs. HUVECs were stimulated with PMA (200 ngml^{-1}, 60 min), ionomycin (IO, 1 µM, 30 min) or melittin (1 µM, 60 min) in **(a)** the presence or absence of the ADAM17/10 inhibitor GW (2 µM), and the preferential ADAM10 inhibitor GI (2 µM) or **(b)** the presence or absence of the broad-spectrum metalloprotease inhibitor marimastat (MM, 10 µM) or the ADAM17-blocking antibody D1 (D1, 200 nM). Cell supernatants were analysed for soluble TNFR1 by ELISA. All stimuli significantly increased TNFR1 shedding (a: n=5; b: n=3;± s.e.m;*P<0.05). Marimastat, the antibody D1 and the ADAM17/10 inhibitor GW (2 µM) significantly reduced the induced proteolysis (a: n=4; b: n=3; ± s.e.m;#P<0.05). N.s.: non-significant.

Supplementary Figure 8

a

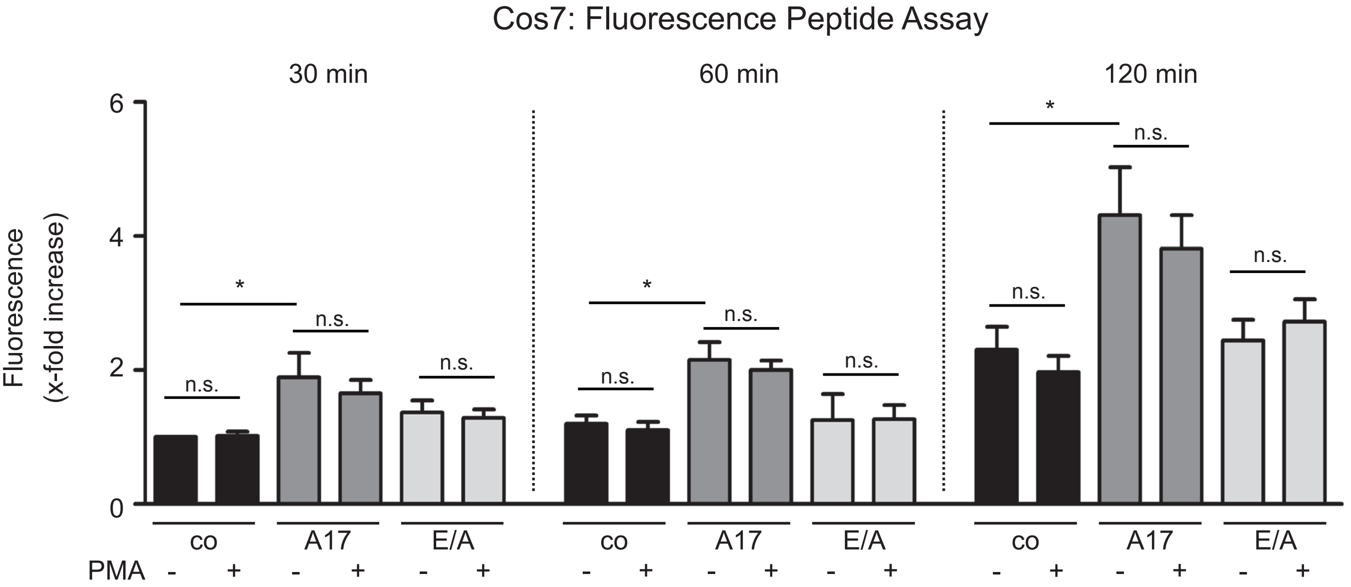

b

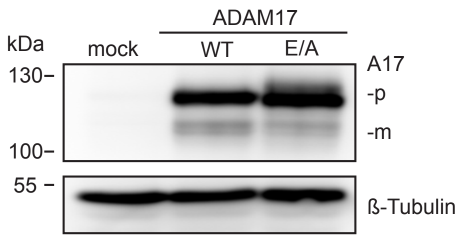

Supplementary Figure 8. PMA does not increase the cell-associated enzyme activity of ADAM17. (a) COS7 cells were transfected with ADAM17-wild-type (A17) or inactive ADAM17-E/A mutant (E/A). Protease cell surface activity was determined for the indicated time points by incubating the cells with a soluble fluorogenic ADAM peptide substrate in the absence or presence of PMA (200 $ngml^{-1}$). ADAM17 overexpression significantly increased peptide cleavage (*n*≥3;± s.e.m;**P*<0.05). The ADAM17 sheddase activator PMA did not affect the enzymatic cell surface activity. The values shown are normalised to the unstimulated mock-transfected COS7 cells (co, 30 min). N.s.: non-significant. **(b)** Transfection efficiency was controlled in parallel by western blot analyses (p = pro, m = mature ADAM17). ß-Tubulin was used as loading control.

Supplementary Figure 9

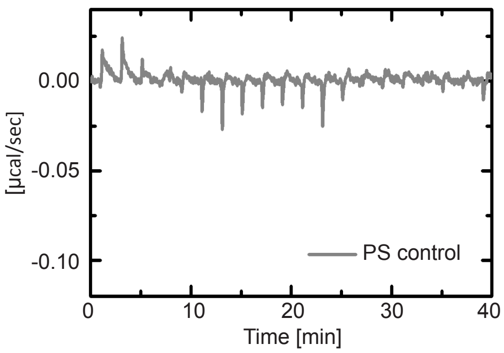

Supplementary Figure 9. Control for isothermal titration calorimetry measurements. As control, buffer (5 mM HEPES) was titrated 20 times into a solution containing PS liposomes and the heat of the interaction was recorded. No reaction could be observed.

Supplementary Figure 10

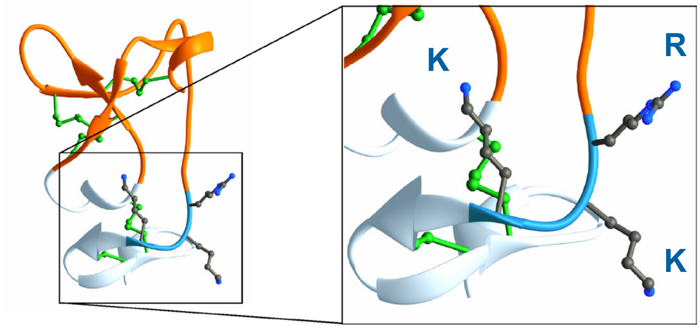

Supplementary Figure 10. Localisation of the PS-binding motif in the flexible part of the MPD. PDI-treatment converts the flexibel unstructured part of the MPD into an unflexible fixed structure. The identified PS-binding motif RK_K is located in this flexible region which is affected by PDI treatment. Ribbon presentation of a representative structure of the closed MPD. The grey and blue coloured part of the molecule is flexible in the open conformation. Disulfide bridges are depicted in green.

Supplementary Figure 11a

Sequence of MPD-3x

1 MGSSHHHHHH SSGLVPRGSH MDDDDKFCER EQQLESCACN ETDNSCKVCC 50

51 RDLSGRCVPY VDAEQKNLFL GGGGPCTVGF CDMNGKCE 100

M Signalpeptide (cleaved in matured protein)

G Mutation sites

Theoretical MW (Da)	
5x S-S	9497.9501
4x S-S	9499.9657
3x S-S	9501.9814
2x S-S	9503.9970
1x S-S	9506.0199
0x S-S	9508.0283

Full MS of intact MPD-3x

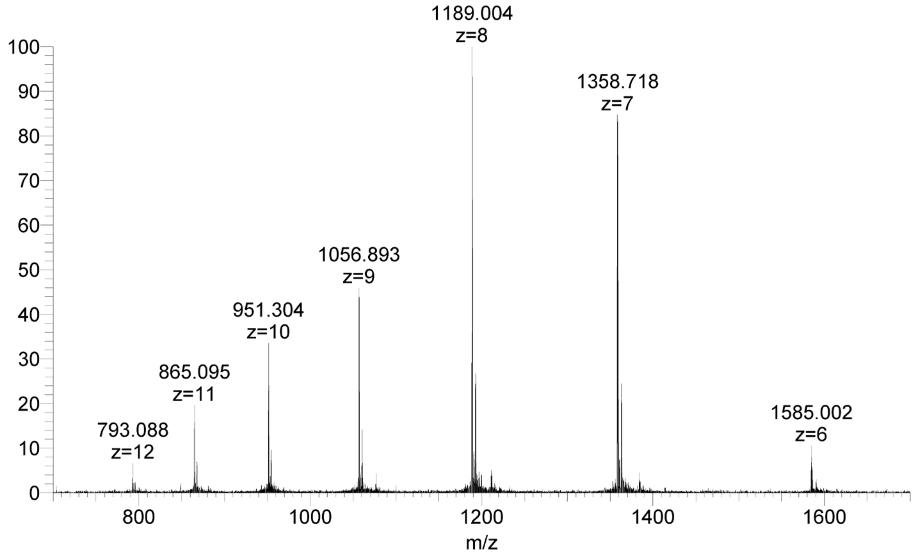

Measured m/z (monoisotopic)	
6+	1584.0033
7+	1357.8574
8+	1188.2555
9+	1056.3346
10+	950.8052

Measured MW (Da)	
6+	9497.9761 [+2.74 ppm]
7+	9497.9509 [+0.08 ppm]
8+	9497.9858 [+3.76 ppm]
9+	9497.9459 [-0.44 ppm]
10+	9497.9792 [3.06 ppm]

Supplementary Figure 11a. Theoretical MWs and acquired MS spectrum of intact MPD-3x. To determine the number of closed disulfide bonds that are present in MPD-3x, the native protein was analyzed by intact protein analysis prior partial reduction and alkylation on a LTQ Orbitrap Velos. In average a mass of 9497.9(7) was calculated by deconvolution, indicating that all disulfide bonds are closed.

Supplementary Figure 11b

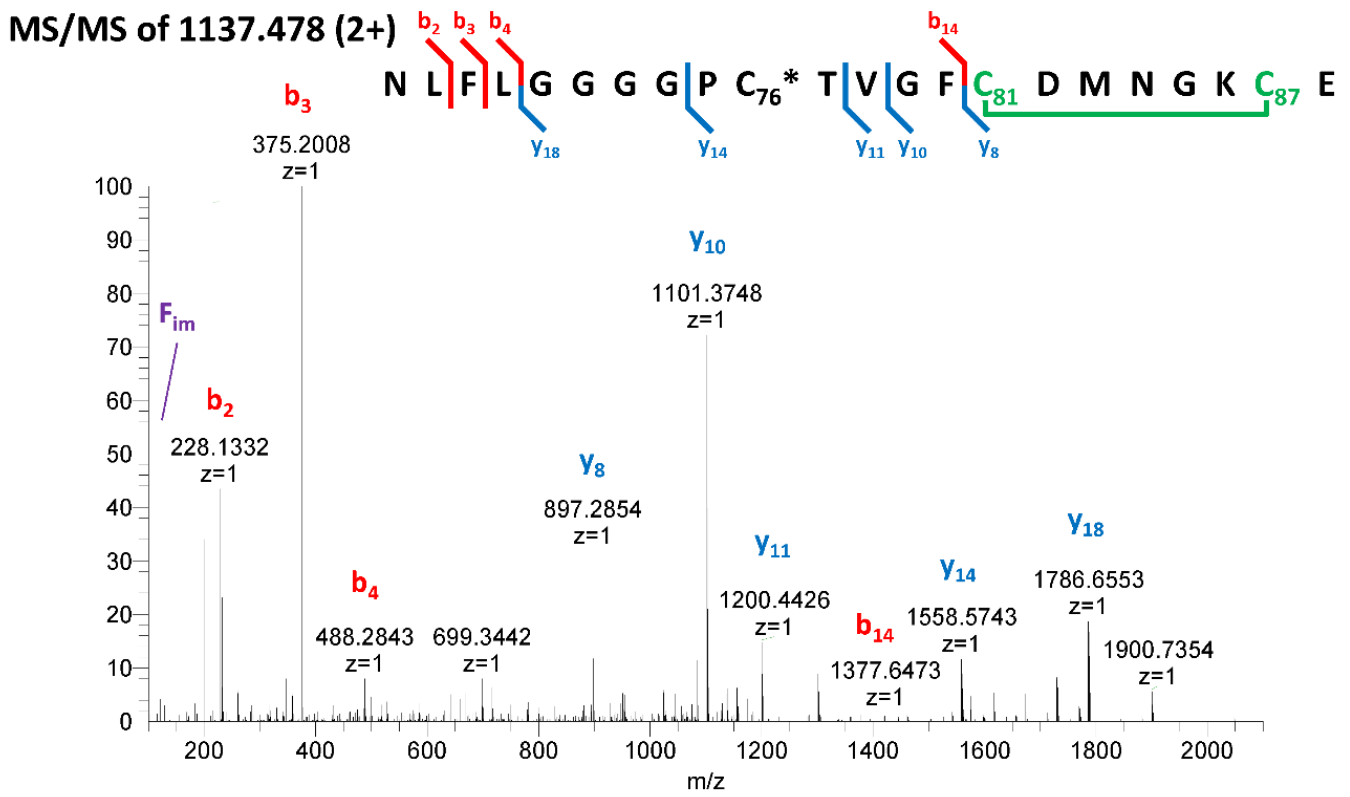

Supplementary Figure 11b. C-terminal peptide of MPD-3x derived from tryptic digestion. HCD-MS/MS spectrum of the doubly-charged peptide ($[M+2H]^{2+}$ at m/z of 1137.478) acquired with a UPLC-Q Exactive Plus MS. Disulfide linkage of Cys81-Cys87 confirms open conformation of the protein. *: alkylated cysteine. Numbering of the cysteines refers to Suppl. figure 11a.

Supplementary Figure 12

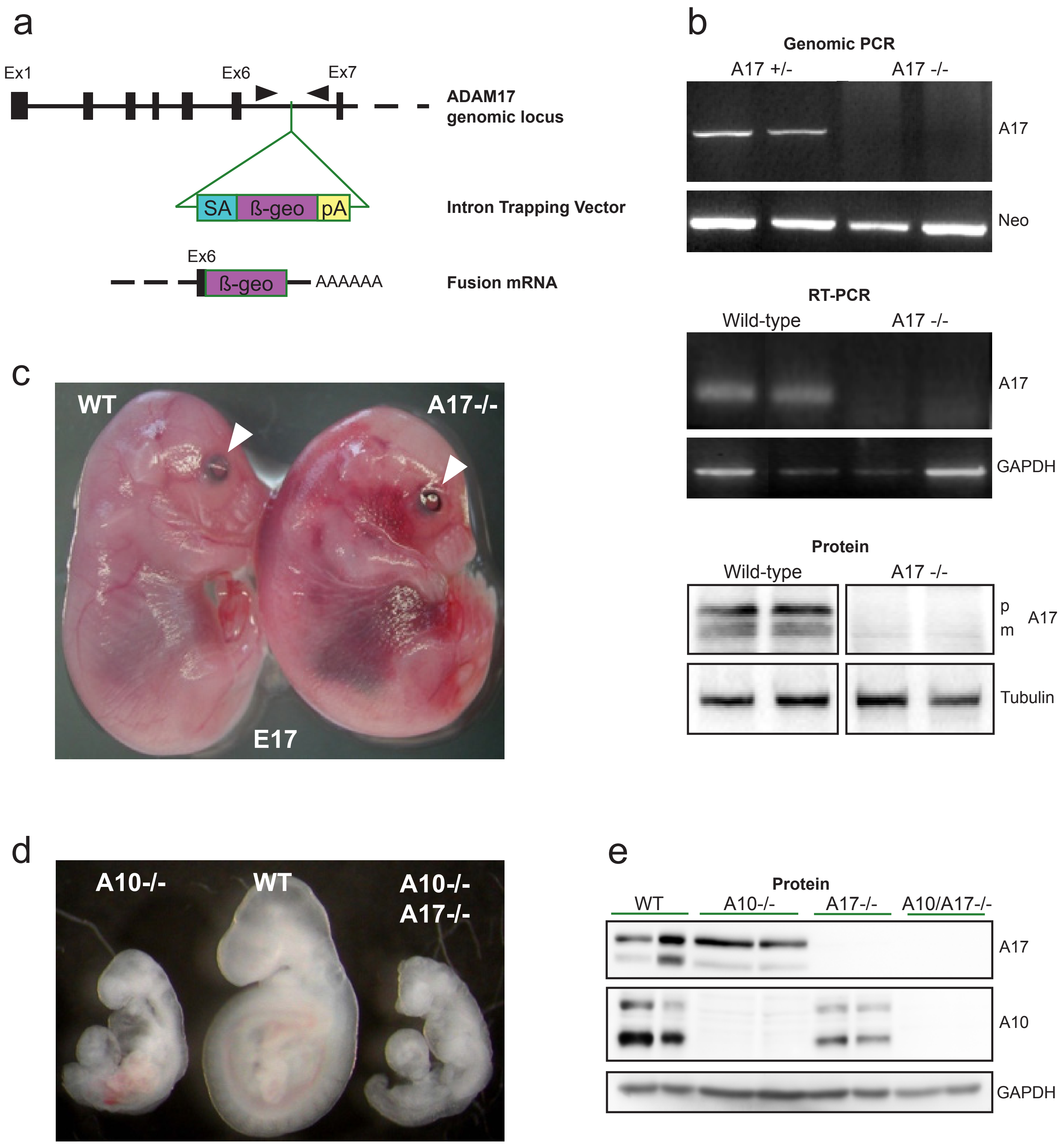

Supplementary Figure 12. Generation of ADAM17-deficient and ADAM10/17 double-deficient mice and cells. **(a)** Scheme representing the wild-type ADAM17 locus and the generation of the ADAM17 gene-trap allele. **(b)** ADAM17 (A17) deficiency was evidenced by PCR, RT-PCR and immunoblot analysis of the embryos. **(c)** The phenotype of embryonically lethal ADAM17-deficient mice is characterized by eye (open eyelids, arrow) and skin defects. **(d)** ADAM10-single and ADAM10/17-double-deficient mice at embryonic day (E) 9. Double-deficient mice reproduce mainly the phenotype of ADAM10-deficient mice (A10-/-). A clear difference of the external phenotype is seen at the level of the forming cerebral vesicles, which are significantly smaller in double-deficient mice. **(e)** Different cell lines derived from single and double-deficient ADAM10/17 embryos were generated and controlled by immunoblot analysis for ADAM-deficiency.

Supplementary Figure 13

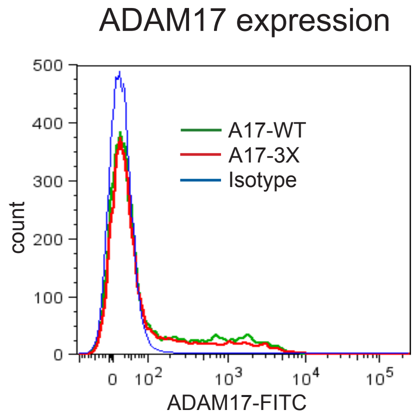

Supplementary Figure 13. Flow cytometric analysis of ADAM17 and ADAM17-3x expression. ADAM10/ADAM17 double-deficient cells were transfected with wild-type ADAM17 (WT-A17) or ADAM17-3x (A17-3x) and analysed by flow cytometry for cell surface expression.

Supplementary Figure 14

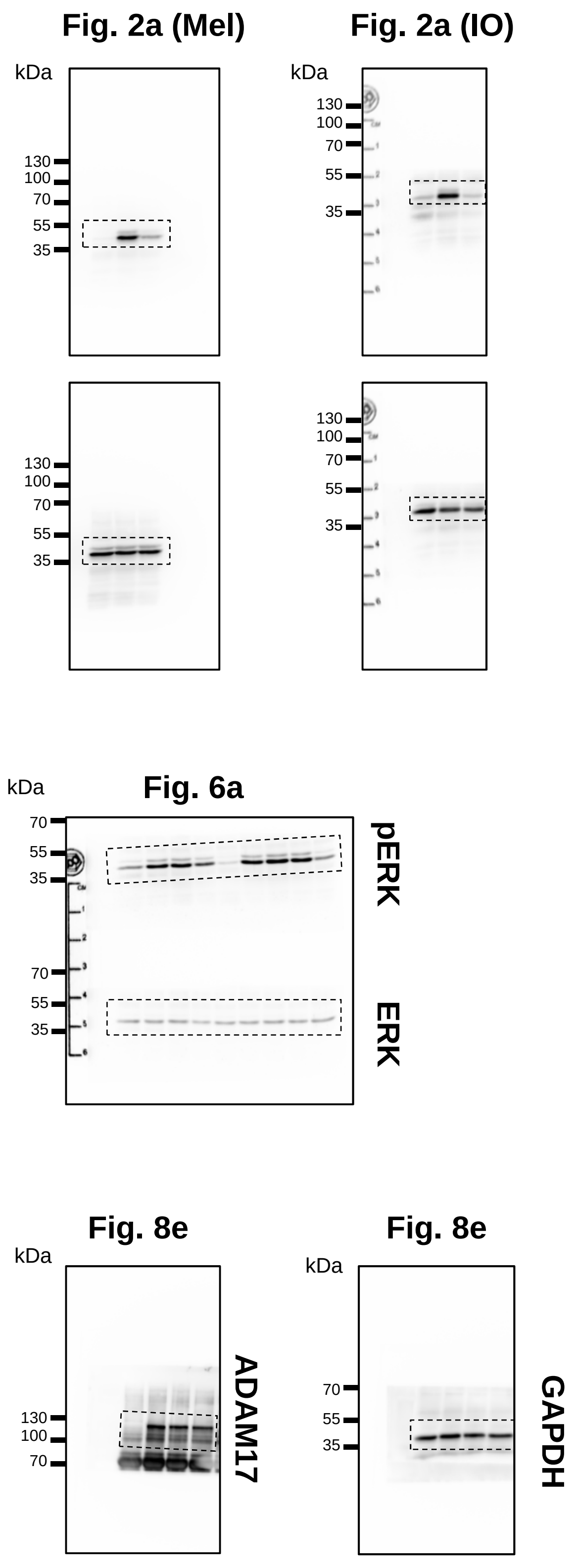

Supplementary Fig. 14. Full images of western blots included in the manuscript.
